# Development and validation of preeclampsia predictive models using key genes from bioinformatics and machine learning approaches

**DOI:** 10.3389/fimmu.2024.1416297

**Published:** 2024-10-31

**Authors:** Qian Li, Xiaowei Wei, Fan Wu, Chuanmei Qin, Junpeng Dong, Cailian Chen, Yi Lin

**Affiliations:** ^1^ Reproductive Medicine Center, Shanghai Sixth People’s Hospital Affiliated to Shanghai Jiao Tong University School of Medicine, Shanghai, China; ^2^ The International Peace Maternity and Child Health Hospital Affiliated to Shanghai Jiao Tong University School of Medicine, Shanghai, China; ^3^ Department of Automation, Shanghai Jiao Tong University, Key Laboratory of System Control and Information Processing, Ministry of Education of China, Shanghai, China

**Keywords:** preeclampsia, machine learning, deep learning, bioinformatics, immune cell infiltration

## Abstract

**Background:**

Preeclampsia (PE) poses significant diagnostic and therapeutic challenges. This study aims to identify novel genes for potential diagnostic and therapeutic targets, illuminating the immune mechanisms involved.

**Methods:**

Three GEO datasets were analyzed, merging two for training set, and using the third for external validation. Intersection analysis of differentially expressed genes (DEGs) and WGCNA highlighted candidate genes. These were further refined through LASSO, SVM-RFE, and RF algorithms to identify diagnostic hub genes. Diagnostic efficacy was assessed using ROC curves. A predictive nomogram and fully Connected Neural Network (FCNN) were developed for PE prediction. ssGSEA and correlation analysis were employed to investigate the immune landscape. Further validation was provided by qRT-PCR on human placental samples.

**Result:**

Five biomarkers were identified with validation AUCs: *CGB5* (0.663, 95% CI: 0.577-0.750), *LEP* (0.850, 95% CI: 0.792-0.908), *LRRC1* (0.797, 95% CI: 0.728-0.867), *PAPPA2* (0.839, 95% CI: 0.775-0.902), and *SLC20A1* (0.811, 95% CI: 0.742-0.880), all of which are involved in key biological processes. The nomogram showed strong predictive power (C-index 0.873), while FCNN achieved an optimal AUC of 0.911 (95% CI: 0.732-1.000) in five-fold cross-validation. Immune infiltration analysis revealed the importance of T cell subsets, neutrophils, and NK cells in PE, linking these genes to immune mechanisms underlying PE pathogenesis.

**Conclusion:**

*CGB5*, *LEP*, *LRRC1*, *PAPPA2*, and *SLC20A1* are validated as key diagnostic biomarkers for PE. Nomogram and FCNN could credibly predict PE. Their association with immune infiltration underscores the crucial role of immune responses in PE pathogenesis.

## 1 Introduction

Preeclampsia (PE) is a common gestational complication characterized by the sudden onset of hypertension (≥140/90 mmHg) and proteinuria (≥0.3 g/24 h) after the 20th week of gestation (1). PE poses a significant threat to maternal and fetal health, accounting for over 70,000 maternal deaths and 500,000 fetal deaths globally each year (2).

Currently, treatment options for different types of PE remain limited. The only definitive cure is to terminate the pregnancy, a measure that can reduce maternal mortality but fails to improve long-term outcomes. The limited effectiveness of current clinical treatments stems from an incomplete understanding of the disease’s pathogenesis, which hampers the development of personalized and precise therapeutic approaches. Recent studies have leveraged sequencing data and bioinformatics analyses to identify reliable key genes that cause the onset of preeclampsia (3–6). Public databases offer vast data for reanalysis and integration, enhancing resource use and increasing sample sizes for more robust analyses (7). Moreover, machine learning algorithms, particularly feature selection techniques, have proven effective in uncovering key information from high-throughput genomic data, identifying pivotal genes influencing disease progression (8, 9). Despite these advances, the clinical practice still lacks sensitive and specific biomarkers for PE, underscoring the need for their identification and validation.

**Figure 1:**
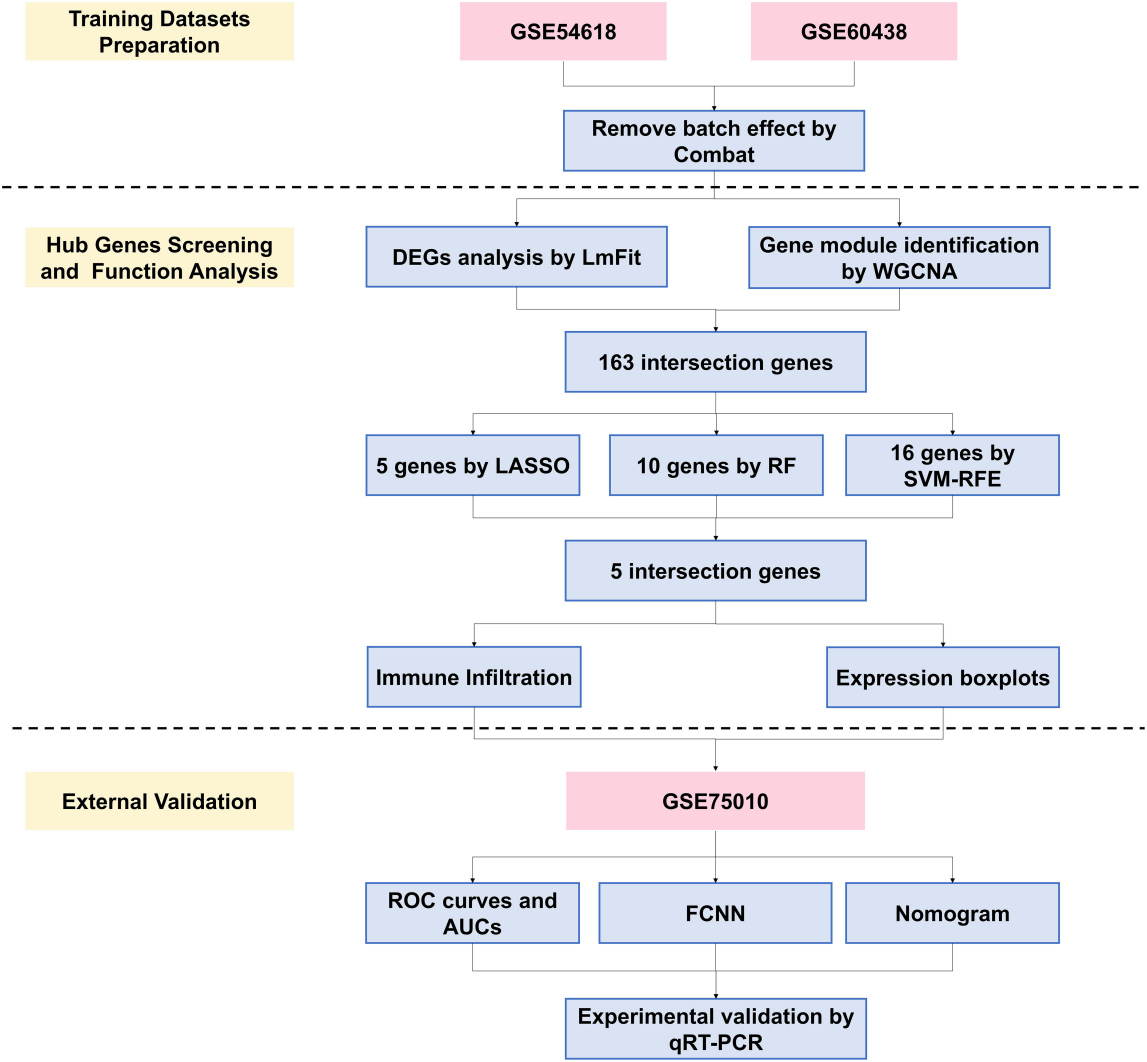
Flowchart of the study. WGCNA, weighted gene co-expression network analysis; DEGs, differentially expressed genes; LASSO, least absolute shrinkage and selection operator; RF, random forest; SVM-RFE, support vector machine recursive feature elimination; ROC, receiver operating characteristic; AUC, area under curve; FCNN, Fully Connected Neural Network; PE, preeclampsia.

This study developed a pipeline to screen and analyze two Gene Expression Omnibus (GEO) datasets as training sets. After removing batch effects, we identified functionally differentially expressed genes (DEGs) using Limma and Weighted Gene Co-Expression Network Analysis (WGCNA) algorithms. The intersection of these DEGs underwent feature selection through machine learning, revealing five critical genes. Functional analysis, including their relationship with immune cell infiltration and validation with an external GEO dataset, highlighted their importance. The constructed nomogram and FCNN prediction models offer new potential for PE diagnosis and therapeutic strategies. The analysis pipeline is depicted in **Figure 1**.

## 2 Methods

### 2.1 Data downloading and study population

The datasets used in this study were downloaded as pre- processed series_matrix.txt.gz files from the GEO, with inclusion criteria being (1). sample origin from placental tissue; (2). gene microarray data; and (3). clear grouping information. Samples without a normal pregnancy control group and blood sample data were excluded. Furthermore, to minimize biases introduced by different gene microarray platforms, datasets originating from the same GPL platform were statistically identified and selected. Ultimately, two databases, GSE54618 and GSE60438, were used as training cohorts and GSE75010 was used for external validation in the study.

The sequencing platform for GSE54618 is the GPL10558, Illumina HumanHT-12 V4.0 expression beadchip, which includes 12 placenta samples with normal blood pressure and 12 samples with preeclampsia. The data from GSE60438 originate from two sequencing platforms, the GPL6884 Illumina HumanWG-6 v3.0 expression beadchip and the GPL10558 Illumina HumanHT-12 V4.0 expression beadchip. To minimize biases, we ultimately selected 77 samples from the GPL10558 platform within GSE60438, comprising 37 samples with normal blood pressure and 40 preeclampsia samples. Additionally, the GSE75010 dataset is based on the GPL6244, Affymetrix Human Gene 1.0 ST Array platform, which includes a total of 80 preeclamptic placentas and 77 non-preclamptic placentas. In summary, this study analyzed a total of 258 samples, including 132 preeclampsia cases and 126 normal samples.

### 2.2 Data preprocessing

The GEOquery package and the ComBat algorithm were employed for the downloading, merging, and preprocessing of the databases. The ComBat algorithm is an empirical Bayes-based method crucial in bioinformatics for correcting batch effects in high-throughput data, particularly gene expression studies (10). It adjusts for technical variations across datasets, ensuring more reliable biological interpretations.

The preprocessing steps included (1). reading GeneIDs and mapping gene probes; (2). removing null probes; (3). addressing multi-probe correspondences; (4). merging samples from the two databases; (5). processing and combining group information; and (6). applying the ComBat algorithm to analyze samples from both sources and remove batch effects.

### 2.3 Differential expression genes analysis

The “LmFit” function from the ‘Limma’ package was used to fit a linear model to the expression dataset and experimental design matrix. A contrast matrix was applied to define comparisons between groups, and the Empirical Bayes method was used to smooth standard errors, improving statistical power in small sample datasets. The datasets used in this study (GSE54618, GSE60438, and GSE75010), were assessed for comparability in key clinical characteristics. Statistical analyses (e.g., t-tests, chi-square tests) were performed to confirm that there were no significant differences between groups for these variables, as mentioned in the original articles. DEGs were identified using a significance threshold of P < 0.05. The log fold change (logFC) threshold was defined as mean(abs(logFC)) + 2*sd(abs(logFC)), where logFC was computed using a base-2 logarithmic transformation. This approach ensures that genes are filtered based on both statistical significance and the magnitude of expression change, thereby improving the robustness of DEG selection. The upregulated and downregulated genes were included in the subsequent analyses because both types of gene expression changes can provide valuable insights into the biological processes and pathways involved. Heatmaps and volcano plots were used to visualize the DEGs. In the volcano plot, p-values were displayed on a log10 scale and logFC on a log2 scale. For this initial step, unadjusted p-values were used to maximize the number of candidate genes, which were subjected to further analysis.

### 2.4 Weight co-expression network analysis

The WGCNA algorithm is another systems biology method used to describe the correlation patterns among genes across microarray samples (11). WGCNA can be used for finding clusters of highly correlated genes, summarizing such clusters using the module eigengene or an intramodular hub gene, relating modules to one another and to external sample traits, and calculating module membership measures.

In the application of WGCNA, the process begins by calculating pairwise correlations between all genes in the dataset from the expression matrix, focusing particularly on genes with variance in the top 25%. This is followed by a rigorous quality control step using the “goodSamplesGenes” function from the WGCNA package. The analysis then involves the identification and exclusion of any outlier samples to ensure the integrity of the data. A crucial step in the process is the calculation and determination of a soft threshold power, which serves to emphasize strong correlations while penalizing weaker ones. Subsequently, the gene-gene correlation matrix is transformed into an adjacency matrix, which is then converted into a Topological Overlap Matrix (TOM) to enhance the robustness of the network by considering not just direct correlations between genes, but also their shared connections. Modules of highly correlated genes are identified using hierarchical clustering methods applied to the TOM. These modules are then correlated with external sample traits to identify those that are associated with specific traits. The final step involves selecting the most strongly correlated gene modules for further in-depth analysis. 

### 2.5 GO and KEGG functional enrichment analysis

Gene Ontology (GO) enrichment and Kyoto Encyclopedia of Genes and Genomes (KEGG) (12) pathway enrichment analyses were conducted to elucidate the primary biological characteristics of the DEGs. A threshold of adjusted P-value < 0.05 was set for statistical significance. The “Padjustedmethod” was set as “BH”. The visualization of the GO enrichment maps, derived from the annotation analysis, was achieved using the “ggplot2” and “GOplot” packages in the R programming environment. To summarize and visualize the enrichment results of the DEGs, we utilized a Treemap (rectangular tree diagram) to display the GO terms. The Treemap groups GO terms based on their parent categories, where each term’s rectangle size is proportional to its enrichment score. This visualization helps to highlight the most significant biological processes by representing them in larger rectangles. The Treemap was generated using the “rrvgo” package, which automatically adjusts the size and color of each rectangle to indicate the relative importance and functional grouping of the terms. Furthermore, enrichment analyses for disease ontology (DO) terms associated with DEGs were performed utilizing the “clusterProfiler” and “DOSE” packages in R (13), providing a comprehensive understanding of the disease-related biological processes and pathways implicated by the DEGs.

### 2.6 Hub genes screening

The gene sets with strong correlations identified through WGCNA were intersected with DEGs to identify candidate hub genes. These candidate genes were then subjected to feature selection using three distinct methods: the least absolute shrinkage and selection operator (LASSO) (14) method, random forest (RF) (15) and the support vector machine recursive feature elimination (SVM-RFE) (16). Each method independently screened for hub genes. The intersection of genes identified by LASSO, RF and SVM-RFE was then taken to determine the final set of hub genes. The specific code implementation process of these three algorithms can be viewed in **Supplementary Material**. This refined list of hub genes serves as the basis for subsequent downstream analyses, ensuring a focused and precise approach to understanding the genetic underpinnings of the study’s specific biological context.

### 2.7 Diagnostic value of the hub genes in external validation cohort

Initially, box plots are created to compare the expression differences of the selected hub genes between the preeclampsia group and the control group in dataset GSE75010. This visual representation provides an immediate and clear comparison of gene expression levels across the two groups. Following this, the predictive efficacy of the hub genes as diagnostic biomarkers for preeclampsia is assessed using Receiver Operating Characteristic (ROC) curves by using the pROC package. The Area Under the Curve (AUC) value is employed as a quantitative standard to evaluate the diagnostic performance of these genes. A higher AUC value indicates a better diagnostic ability of the hub genes to distinguish between preeclampsia and normal conditions, thereby validating their potential as effective biomarkers in preeclampsia screening.

### 2.8 Construction and verification of diagnostic prediction models for preeclampsia

Nomogram is a common method for visualizing logistic regression prediction models. The ‘rms’ package was employed to develop a logistic regression model, with five key genes as independent variables, aiming to predict the binary outcome of PE versus non-preeclampsia (nonPE). To enhance the model’s reliability and adjust for potential overfitting, a calibration curve was constructed using the bootstrap resampling method with 1000 replications (B=1000) (17). The concordance index (C-index), indicative of predictive accuracy, was assessed both before and after calibration. Generally, a C-index from 0.50 to 0.70 denotes low accuracy, 0.71 to 0.90 indicates moderate accuracy, and above 0.90 signifies high accuracy (18).

Moreover, the FCNN, also known as Multilayer Perceptron (MLP), is an artificial neural network architecture. The model was constructed using Python packages, primarily TensorFlow and Keras, along with Scikit-learn for data handling and evaluation. Five-fold cross-validation was employed to assess model performance on a standardized training dataset. The model architecture included a hidden layer with 64 neurons using the ReLU activation function and L2 regularization, followed by an output layer with one neuron utilizing the sigmoid activation function for binary classification. The model was compiled with the Adam optimizer (learning rate set to 0.0001) and binary crossentropy as the loss function. Training occurred over 100 epochs, incorporating early stopping to mitigate overfitting. Model performance was evaluated using ROC curve for both training and validation sets, with AUC values recorded for each fold.

### 2.9 Immune cell infiltration analysis

ssGSEA (Single Sample Gene Set Enrichment Analysis) and GSVA (Gene Set Variation Analysis) were used to analysis immune infiltration in the context of gene expression data (19). The “geneSet.csv” which contains markers for 28 common immune cell types was downloaded online. First, the gene expression data is prepared and normalized. The GSVA package’s ‘gsva’ function can be used in the ‘ssGSEA’ mode to calculate enrichment scores for each immune cell type in each sample, reflecting the relative presence and activity of these cells. The output from both analyses provides a comprehensive view of the immune landscape, revealing the immune cell composition and activity within the samples. 

### 2.10 Tissue collection and quantitative real-time PCR analysis

Placental tissues were immediately collected after delivery or cesarean section, and subsequently rinsed with sterile PBS (HyClone) for a brief period. Total RNA extraction was performed using TRIzol reagent (Life Technologies), followed by cDNA synthesis with the PrimeScript RT reagent kit (Takara). Quantitative real-time PCR (qRT-PCR) analyses were conducted utilizing an SYBR Green Kit (Takara), with ACTIN serving as the internal control. The relative mRNA expression levels were quantified employing the 2^−ΔΔCT^ method. Each sample’s qRT-PCR was replicated across three independent experiments. The metadata of all samples are summarized in **Table 1**, including factors such as maternal age, body mass index (BMI), gravidity, parity, systolic blood pressure (SBP), diastolic blood pressure (DBP), fetal birth weight (FBW), and gestational week (GW). The primer sequences employed are delineated in **Supplementary Table 1**. Statistical comparisons between groups were conducted using Student’s t-test for continuous variables and the chi-square test for categorical variables. Results were expressed as mean ± standard error of the mean (SEM) for continuous variables and as percentages for categorical variables. A significance threshold of P < 0.05 was established for determining significant differences.

**Table 1:** Metadata of samples in qRT-PCR analysis.

Patient characteristics^a^		PE	Control	*P* value^b^
**Age, mean (SD)**		33.8±3.6	31.9±3.6	0.217
**BMI, mean (SD)**		23.2±3.9	22.1±2.4	0.421
**Gravidity, n (%)**	**multi-**	5 (41.7)	8 (61.5)	0.553
	**primi-**	7 (58.3)	5 (38.5)	
**Parity, n (%)**	**multi-**	6 (50.0)	6 (46.2)	1.000
	**primi-**	6 (50.0)	7 (53.8)	
**Gestational Week, mean (SD)**		36.2±1.4	38.8±0.8	**<0.001**
**SBP, mean (SD)**		143.8±17.0	116.9±14.8	**<0.001**
**DBP, mean (SD)**		94.4±10.5	70.6±6.9	**<0.001**
**Fetal Birth Weight, mean (SD)**		2603.3±713.4	3373.8±340.1	**0.004**

^a^Data are presented as mean ± SEM or n (%).

^b^Student’s t-test or chi-square test was used for comparisons, with bold values indicating statistical significance (p < 0.05).

## 3 Results

### 3.1 Batch effect processing

**Figure 2:**
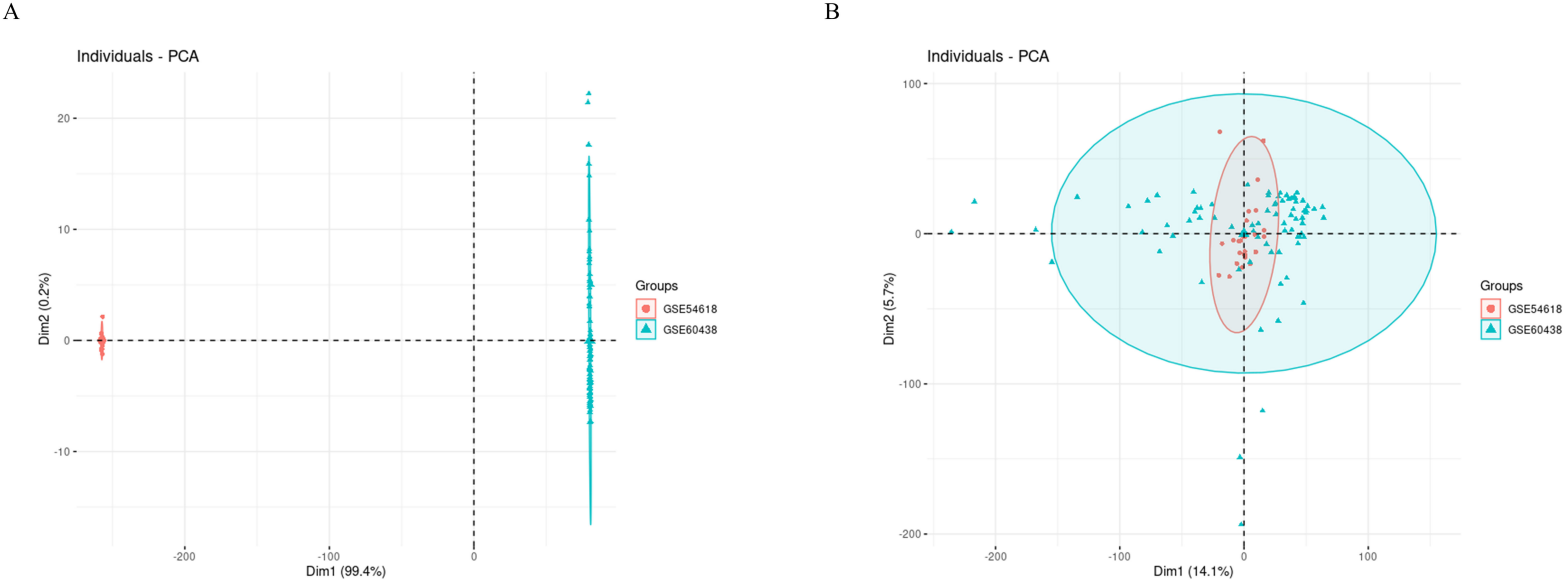
PCA cluster diagrams visualize the effects before and after adjusting for batch differences. **(A)** Raw data before removing batch effect; **(B)** After removing batch effect by ComBat algorithm.

Initially, we employed PCA plots to inspect the presence of batch effects in the two training datasets (**Figure 2A**). A clear batch effect was indeed observed, necessitating the use of the “sva” package to mitigate this bias. After the application of the ComBat algorithm to remove the batch effects, no significant differences were apparent (**Figure 2B**).

### 3.2 DEGs and functional analysis

**Figure 3:**
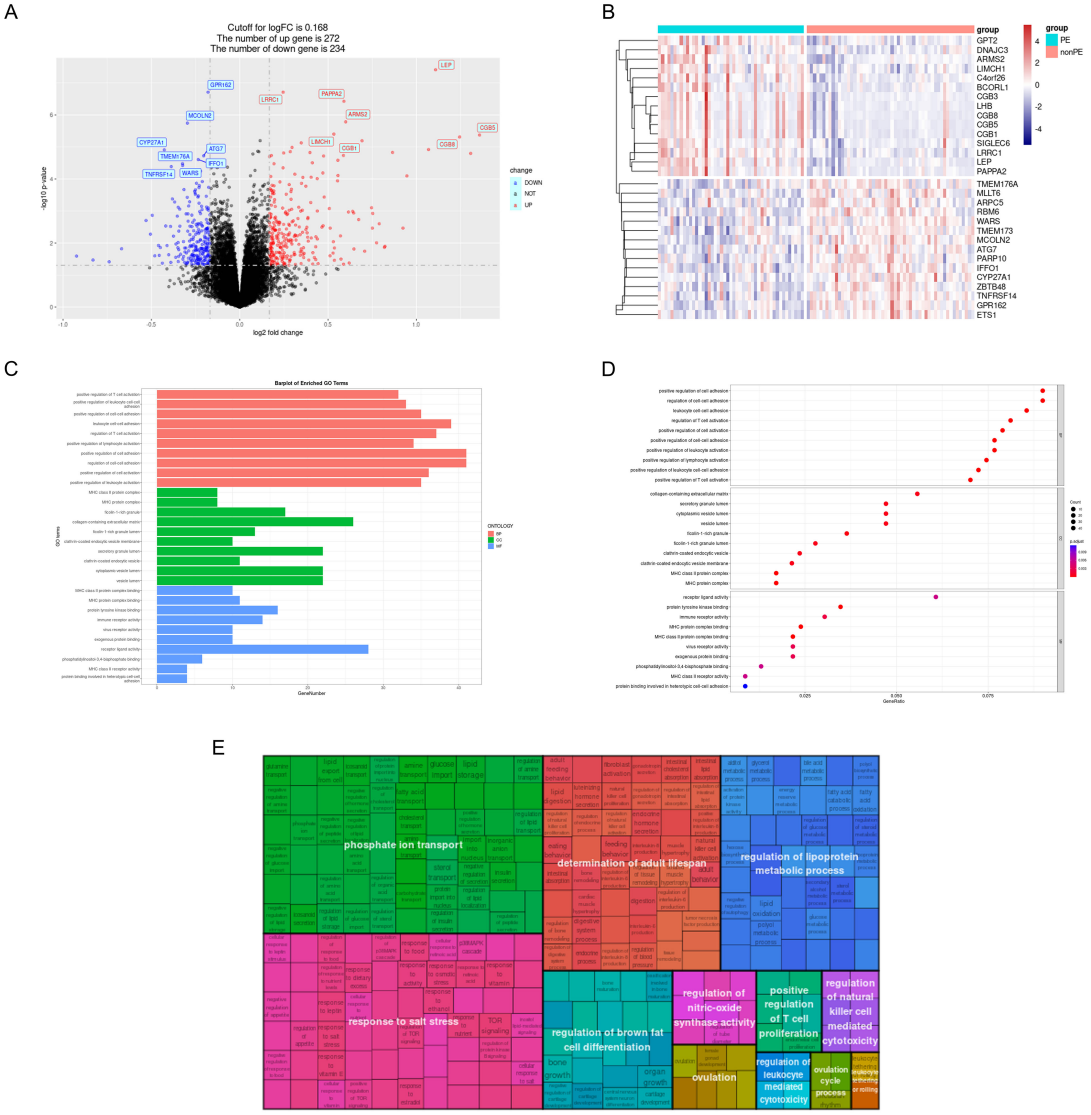
GO analysis of DEGs. **(A)** Volcano plot; **(B)** DEGs heatmap; **(C)** DEGs were represented by bar plots displaying GO enrichment analysis; **(D)** DEGs were represented by dot plots displaying GO enrichment analysis; **(E)** Rectangular tree diagram displayed simplified GO enrichment analysis results. PE, preeclampsia; nonPE, healthy control; GO, Gene Ontology; DEGs, different expressed genes.

A total of 506 DEGs were identified with statistical significance (P<0.05) and a fold change greater than 0.168, comprising 272 up-regulated genes and 234 down-regulated genes (**Figure 3A** and **Supplementary Table S2**). DEGs heatmap also displayed (**Figure 3B**). Subsequently, GO enrichment analysis and KEGG pathway enrichment analysis were conducted to elucidate the functional roles of these DEGs. The results of GO analysis revealed that the DEGs are predominantly associated with T-cell activation, leukocyte adhesion, ficolin-1-rich granule, and MHC (Major Histocompatibility Complex) protein complex binding (**Figures 3C, D**). To further simplify the enrichment results, the simplified results were displayed with a rectangular tree diagram (**Figure 3E**). In this diagram, GO terms are grouped by their parent categories, with the size of each rectangle indicating the enrichment score for that term. Larger rectangles correspond to GO terms associated with a higher number of DEGs or greater statistical significance.

**Figure 4:**
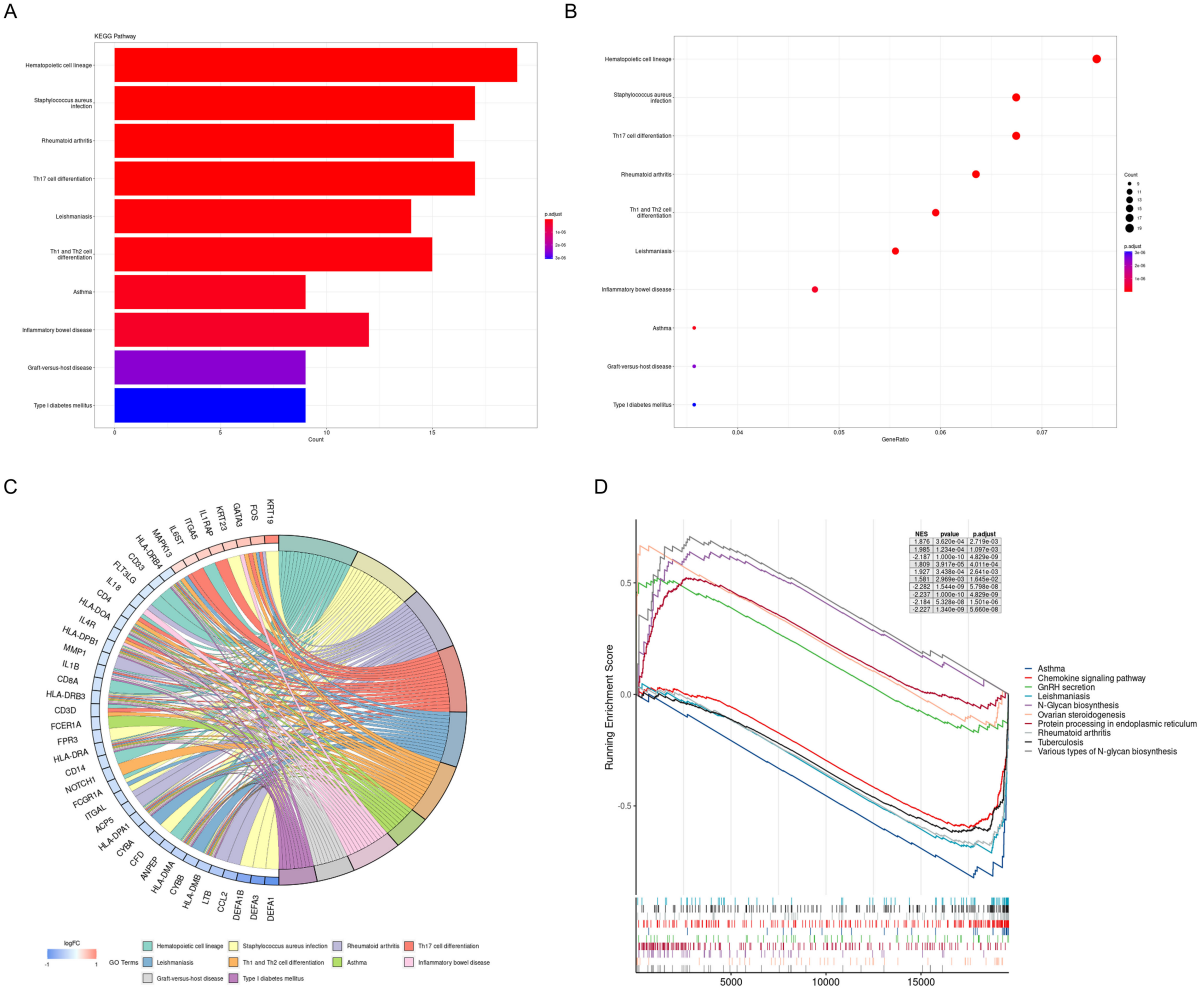
KEGG pathway analysis and GSEA of DEGs. **(A)** DEGs were represented by bar plots displaying results of KEGG pathway analysis; **(B)** DEGs were represented by dot plots displaying results of KEGG pathway analysis; **(C)** Chord diagram displays the key genes; **(D)** 5 most notable upregulation and 5 most notable downregulation pathways enriched by GSEA. KEGG, Kyoto Encyclopedia of Genes and Genomes; GSEA, gene set enrichment analysis.

The results of the KEGG analysis were visualized using a bar plot and bubble chart, while key genes were illustrated through a chord diagram (**Figures 4A–C**). This visualization indicated that the KEGG pathways related to PE predominantly involve hematopoietic cell lineage, rheumatoid arthritis, Th17 cell differentiation, Th1 and Th2 cell differentiation, asthma, and inflammatory bowel disease. Furthermore, a comprehensive GSEA was conducted on the entire gene set, revealing that the most significantly upregulated pathways include GnRH secretion, N-Glycan biosynthesis, ovarian steroidogenesis, protein processing in the endoplasmic reticulum, and various types of N-glycan biosynthesis. In contrast, the pathways experiencing the most pronounced downregulation were asthma, chemokine signaling pathway, leishmaniasis, rheumatoid arthritis, and tuberculosis (**Figure 4D**).

### 3.3 Results of WGCNA analysis

**Figure 5:**
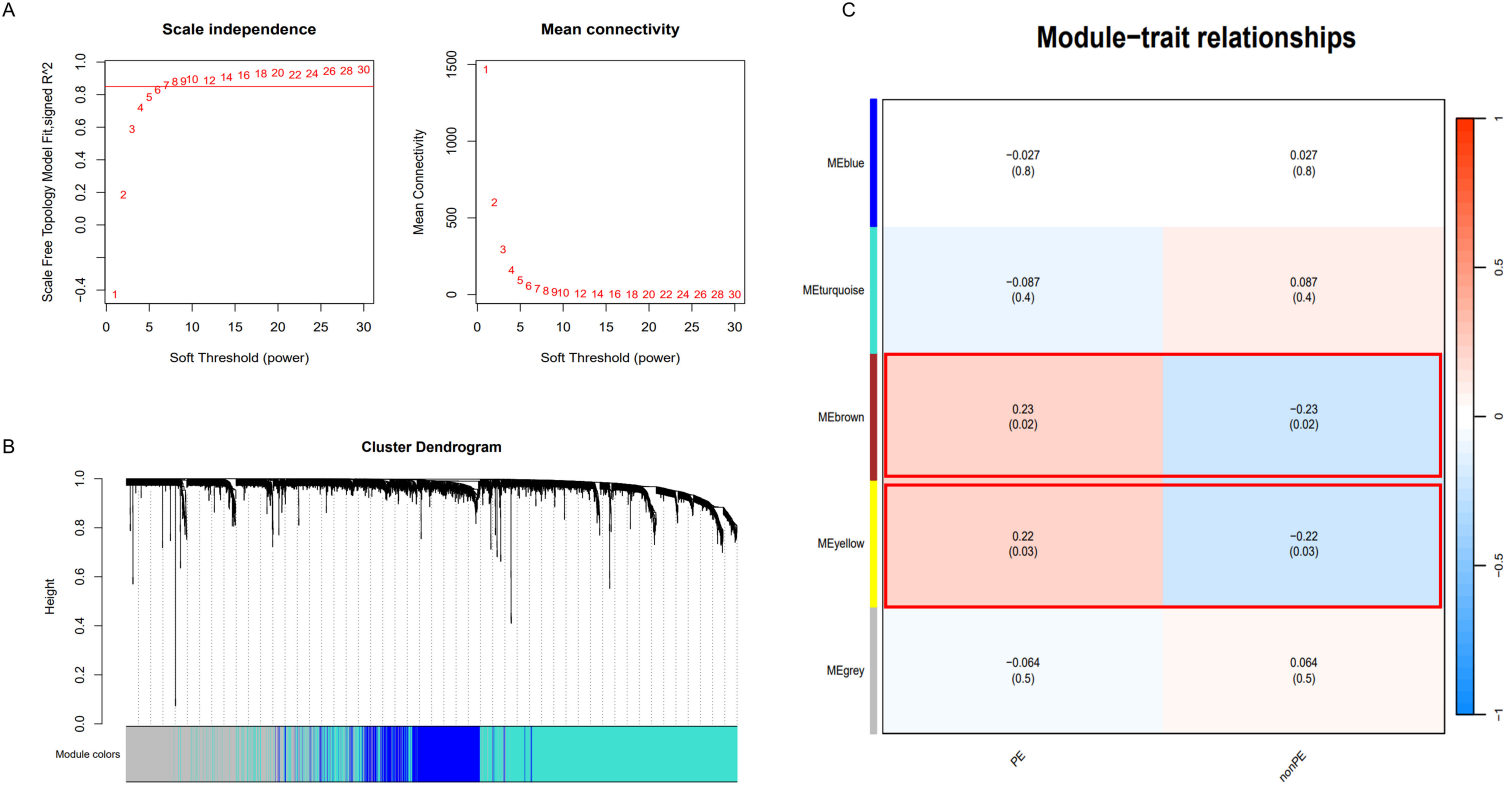
WGCNA analysis. **(A)** Determination of soft-threshold power; **(B)** Cluster dendrogram of highly connected genes in key modules.; **(C)** Relationships between modules and traits in PE. Correlations and P values are included in each cell; PE, preeclampsia; nonPE, healthy control.

Additionally, apart from analyzing differential expression genes, this study employed WGCNA. Using the soft-thresholding approach, a co-expression network was constructed. The parameter β played a crucial role in ensuring that co-expression networks maintained a scale-free topology. A fit index greater than 0.85 was deemed indicative of a scale-free topology, and in this study, β was set at 14 (**Figure 5A**) Furthermore, **Figure 5B** illustrates the hierarchical clustering constructed using the TOM dissimilarity measure.

In the end, a total of 5 co-expression modules were obtained (**Figure 5C** and **Supplementary Tables S3**–**S7**). Among them, the modules with a statistical significance of P<0.05 were considered key modules. It is noteworthy that the “MEbrown” module (correlation coefficient 0.23, p=0.02) and the “MEyellow” module (correlation coefficient 0.22, p=0.03) displayed strong correlations with both positive and negative values. The “MEbrown” module comprised 337 genes, while the “MEyellow” module consisted of 147 genes. In total, 484 genes were collectively utilized for subsequent analysis.

### 3.4 Potential diagnostic genes identification based on machine learning methods

**Figure 6:**
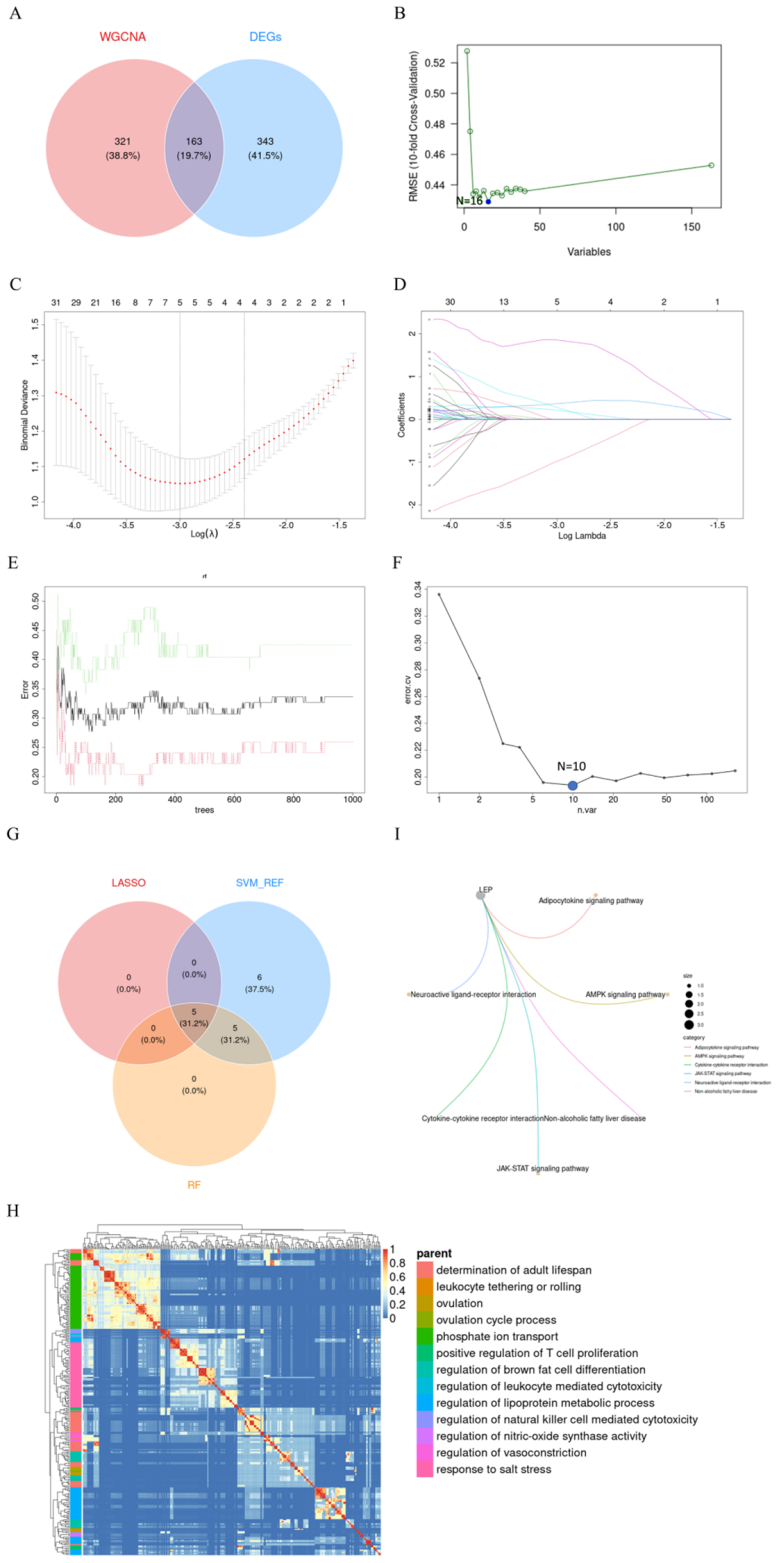
Screening of Hub Genes. **(A)** Crosstalk genes identified from two WGCNA models and DEGs; **(B)** Sixteen genes selected by SVM-RFE with the lowest 5xCV error; **(C, D)** Coefficient profile plot of the LASSO model for PE, showing the final parameter selection λ (lambda), with the upper x-axis representing the number of feature genes; **(E)** Error plot displaying the error rates for different numbers of trees; **(F)** Ten genes selected by RF with the lowest 5xCV error; **(G)** Crosstalk genes identified by LASSO, SVM-RFE, and RF; **(H)** Heatmap showing the results of GO enrichment analysis for five hub genes; **(I)** Six KEGG pathways enriched by *LEP*. PE, preeclampsia; LASSO, Least Absolute Shrinkage and Selection Operator; SVM-RFE, Support Vector Machine-Recursive Feature Elimination; RF, Random Forest; KEGG, Kyoto Encyclopedia of Genes and Genomes; *LEP*, Leptin.

The intersection of 506 DEGs identified using the limma method and 484 genes obtained through WGCNA resulted in 163 overlapping genes (**Figure 6A**). Subsequently, these 163 genes were subjected to feature selection and selection using three distinct algorithms: the SVM-RFE, LASSO, and RF. In the SVM-RFE model, all genes were included for computation, ultimately identifying 16 genes with the lowest 10-fold cross-validation (CV) Root Mean Square Error (RMSE) (**Figure 6B**). Meanwhile, in the LASSO model, with a lambda (λ) value of 0.0499, five genes were identified with non-zero coefficients (**Figures 6C, D**). On the other hand, the RF algorithm effectively identified 10 signature genes by selecting the smallest cross-validation error (**Figures 6E, F**). The intersection of genes post-feature selection by these three methods yielded 5 hub genes: *CGB5*, *LEP*, *LRRC1*, *PAPPA2*, and *SLC20A1* (**Figure 6G**).

Enrichment analyses utilizing GO and Kyoto Encyclopedia of Genes and Genomes KEGG frameworks have yielded a deeper insight into the biological processes and pathways implicated by these five signature biomarkers. The GO analysis indicates that these genes predominantly participate in processes such as the determination of adult lifespan, leukocyte tethering or rolling, ovulation, ovulation cycle process, phosphate ion transport, positive regulation of T cell proliferation, regulation of brown fat cell differentiation, regulation of leukocyte-mediated cytotoxicity, regulation of lipoprotein metabolic process, regulation of natural killer cell-mediated cytotoxicity, regulation of nitric oxide synthase activity, and response to salt stress (**Figure 6H**). Concurrently, the KEGG analysis identified six pathways significantly enriched by these genes, namely the *LEP*, Adipocytokine signaling pathway, AMPK signaling pathway, Cytokine-cytokine receptor interaction, JAK-STAT signaling pathway, Neuroactive ligand-receptor interaction, and Non-alcoholic fatty liver disease (**Figure 6I**).

### 3.5 Diagnostic value and external validation of 5 hub genes in preeclampsia

**Figure 7:**
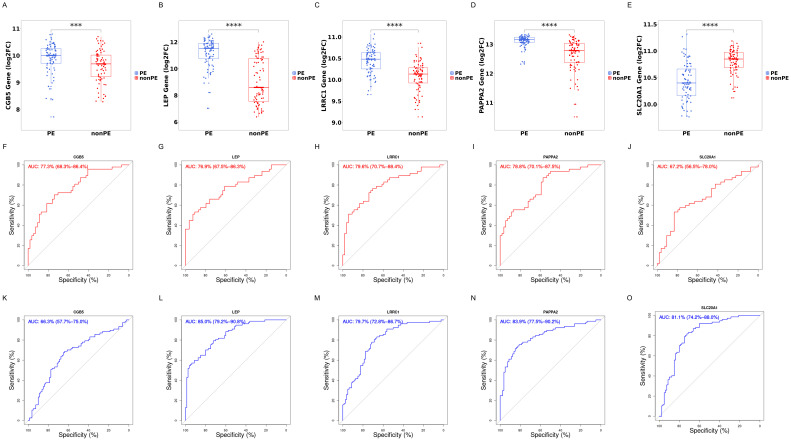
Boxplot and ROC of 5 hub genes. **(A–E)** Gene expression levels of *CGB5, LEP, LRRC1, PAPPA2*, and *SLC20A1* respectively; **(F–J)** ROC curves of *CGB5, LEP, LRRC1, PAPPA2*, and *SLC20A1* in training cohorts; **(K–O)** ROC curves of *CGB5, LEP, LRRC1, PAPPA2*, and *SLC20A1* in validation cohort; PE, preeclampsia; nonPE, healthy control;. *** p < 0.001, **** p < 0.0001.

Subsequently, the diagnostic value of these 5 hub genes was screened. Initially, their expression levels in the training cohort were calculated and displayed using box plots. It was observed that *CGB5*, *LEP*, *LRRC1*, *PAPPA2* showed a significant increase in expression in the PE group (P<0.0001), while the expression of *SLC20A1* significantly decreased in the PE group (P<0.01) (**Figures 7A–E**).

Next, to assess the specificity and sensitivity of these genes for the diagnosis of preeclampsia, ROC curves were conducted for each of the 5 genes. These genes demonstrated commendable diagnostic efficacy in both training and external validation cohorts. In the training cohort, AUC values were as follows: *CGB5* (AUC=0.773, 95%CI: 0.683-0.864), *LEP* (AUC=0.769, 95%CI: 0.675-0.863), *LRRC1* (AUC=0.796, 95%CI: 0.707-0.884), *PAPPA2* (AUC=0.788, 95%CI: 0.701-0.875), and *SLC20A1* (AUC=0.672, 95%CI: 0.565-0.78) (**Figures 7F–J**). In the external validation cohort, AUC values were as follows: *CGB5* (AUC=0.663, 95%CI: 0.577-0.750), *LEP* (AUC=0.850, 95%CI: 0.792-0.908), *LRRC1* (AUC=0.797, 95%CI: 0.728-0.867), *PAPPA2* (AUC=0.839, 95%CI:0.775-0.902), and *SLC20A1* (AUC=0.811, 95%CI: 0.742-0.880) (**Figures 7K–O**).

**Figure 8:**
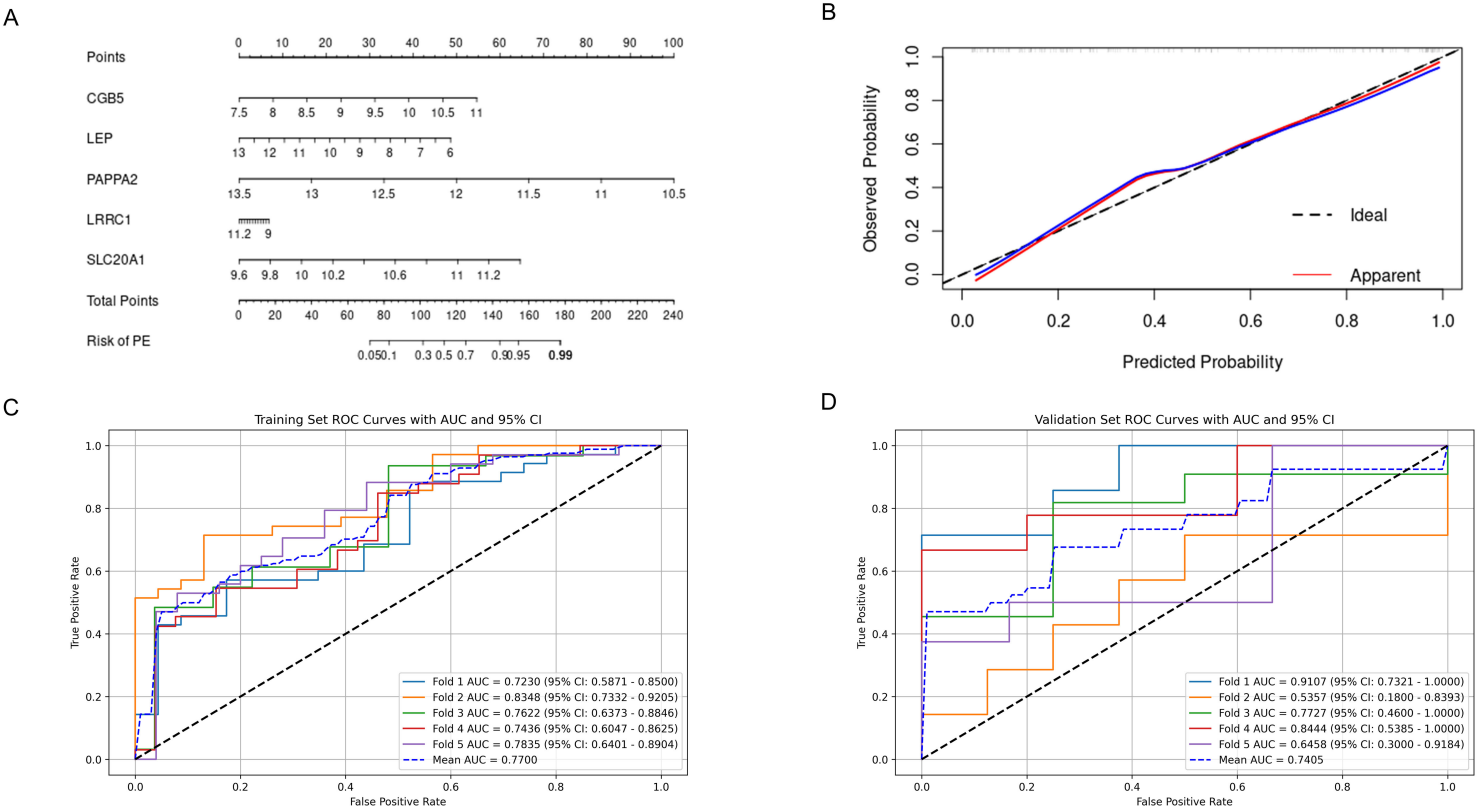
Predictive Performance of the Nomogram and FCNN Model for Preeclampsia. **(A)** Nomogram for predicting preeclampsia; **(B)** Calibration curves assessing the predictive accuracy of the nomogram; **(C)** ROC curve and AUC for the FCNN model on the 5-fold cross-validated training set; **(D)** ROC curve and AUC for the FCNN model on the 5-fold cross-validated validation set. ROC, receiver operating characteristic; AUC, area under the curve; FCNN, fully connected neural network.

The nomogram, incorporating the expression profiles of five pivotal hub genes, serves as an intuitive graphical representation that converts gene expression data into a predictive probability of developing PE (**Figure 8A**). Calibration plots derived from the nomogram model demonstrate a remarkable alignment, signifying an excellent concordance between the nomogram’s predicted probabilities and the observed outcomes (**Figure 8B**). The model exhibits a C-index of 0.887, indicative of moderate predictive accuracy. After bias correction through calibration, the C-index stands at 0.873, reinforcing the model’s robust predictive performance.

Considering that genes often do not function in isolation but interact extensively, a simple linear model may not adequately represent these interactions. Therefore, the use of neural networks for constructing predictive models is considered, and their efficiency is compared with that of nomograms. A FCNN model, comprising one hidden layer with 64 neurons, was constructed using the five hub genes. Furthermore, to reduce overfitting, we employed five-fold cross-validation. ROC curves were used to evaluate the overall diagnostic efficacy of the FCNN model. The mean AUC for the training set was 0.770, and for the validation set, it was 0.741 (**Figures 8C, D**).

### 3.6 Immune infiltration analysis of 5 hub genes

**Figure 9:**
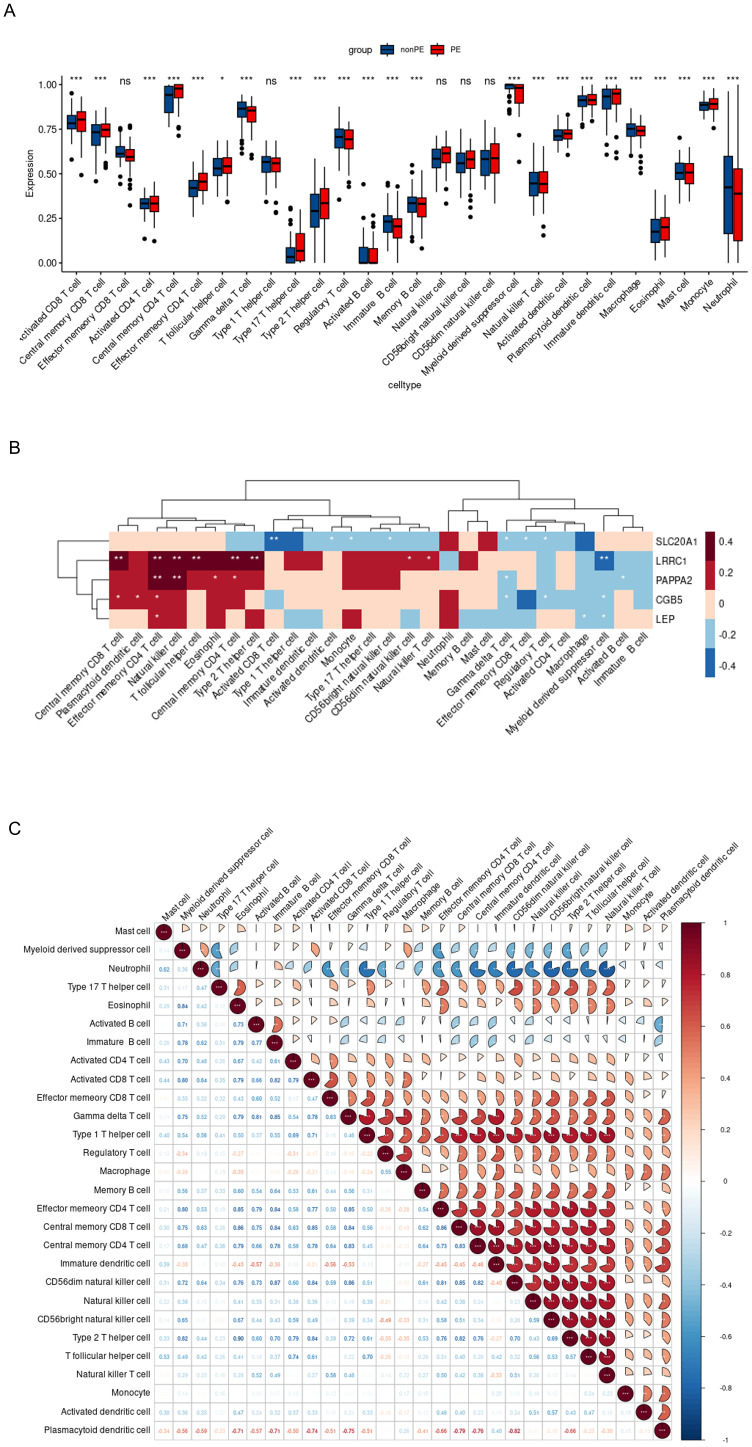
Immune infiltration analysis of 5 hub genes. **(A)**. Evident difference in immune cell types shown by boxplot; **(B)**. Relationship between the expression of 5 hub genes and immunity in PE patients; **(C)**. Correlation map revealed the relationship of the 28 immune cell types; PE, preeclampsia; nonPE, healthy control;. * p < 0.05, *** p < 0.001.

A boxplot analysis was conducted on the expression levels of 28 immune cell types (**Figure 9A**). The findings indicated a decrease in the expression of Myeloid-derived suppressor cells (MDSC), γδ T cells, and NKT cells. In contrast, CD4+ T cells, CD8+ T cells, and Th17 cells, showed an increase in expression. Additionally, the expression of Th17 cells was upregulated while that of Treg cells decreased. These observations underscore the importance of the Th17/Treg balance in the development and progression of preeclampsia. Notably, NK cells, including CD56dim NK and CD56bright NK, did not exhibit significant statistical changes.

**Figure 10:**
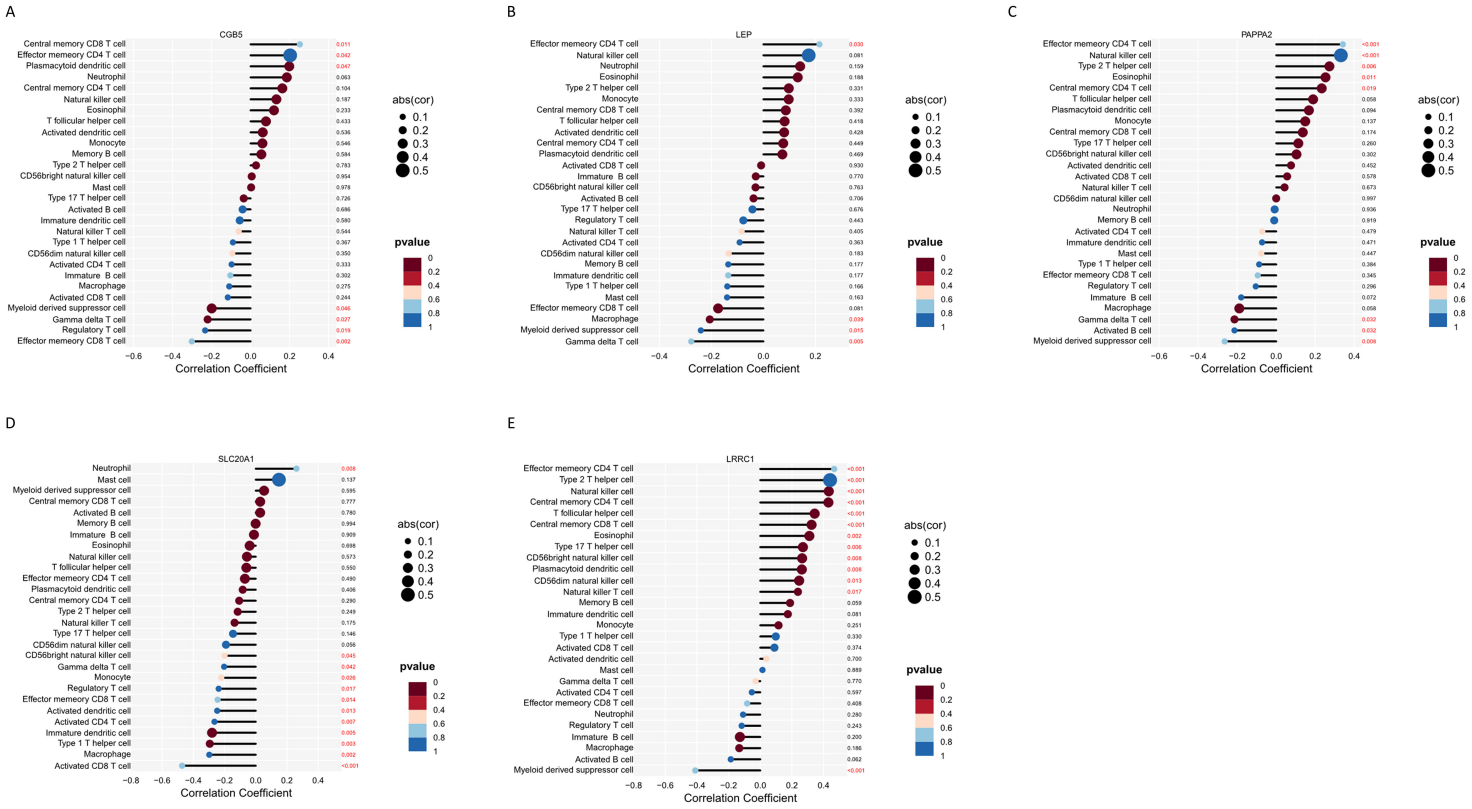
Immune infiltration analysis of 5 hub genes. **(A–E)** Correlation analysis between feature genes and immune cells.

Furthermore, the relationship between the five hub genes and 28 types of immune cells was analyzed (**Figure 9B**). *CGB5* was primarily associated with an increased expression of “Central memory CD8 T cells,” “Plasmacytoid dendritic cells,” and “Effector memory CD4 T cells,” while it was linked to a decreased expression of “Treg” and “Myeloid-derived suppressor cells.” *LEP* mainly exhibited a decreased expression of “Macrophages” and “Myeloid-derived suppressor cells.” *LRRC1* showed a primary connection with an increased expression of “NK cells” and various effector memory cells, along with a decreased expression of “Myeloid-derived suppressor cells.” *PAPPA2* was primarily associated with an increased expression of various effector memory cells (**Figures 10A–E**). Furthermore, correlation analysis of the 28 types of immune cells revealed intriguing results. Activated B cells, immature B cells, mast cells, monocytes, type 17 T helper cells, and eosinophils demonstrated weak or negligible correlations with other immune cell types. In contrast, the majority of T cell subsets, neutrophils, and NK cells exhibited strong positive correlations with other immune cell types, highlighting their significant roles in the pathogenesis of PE. Notably, only neutrophils were found to have a negative correlation with the rest of the immune cell types, suggesting a unique function for this specific immune cell population in the context of PE (**Figure 9C**).

### 3.7 Experimental validation of 5 hub genes by qRT-PCR

**Figure 11:**
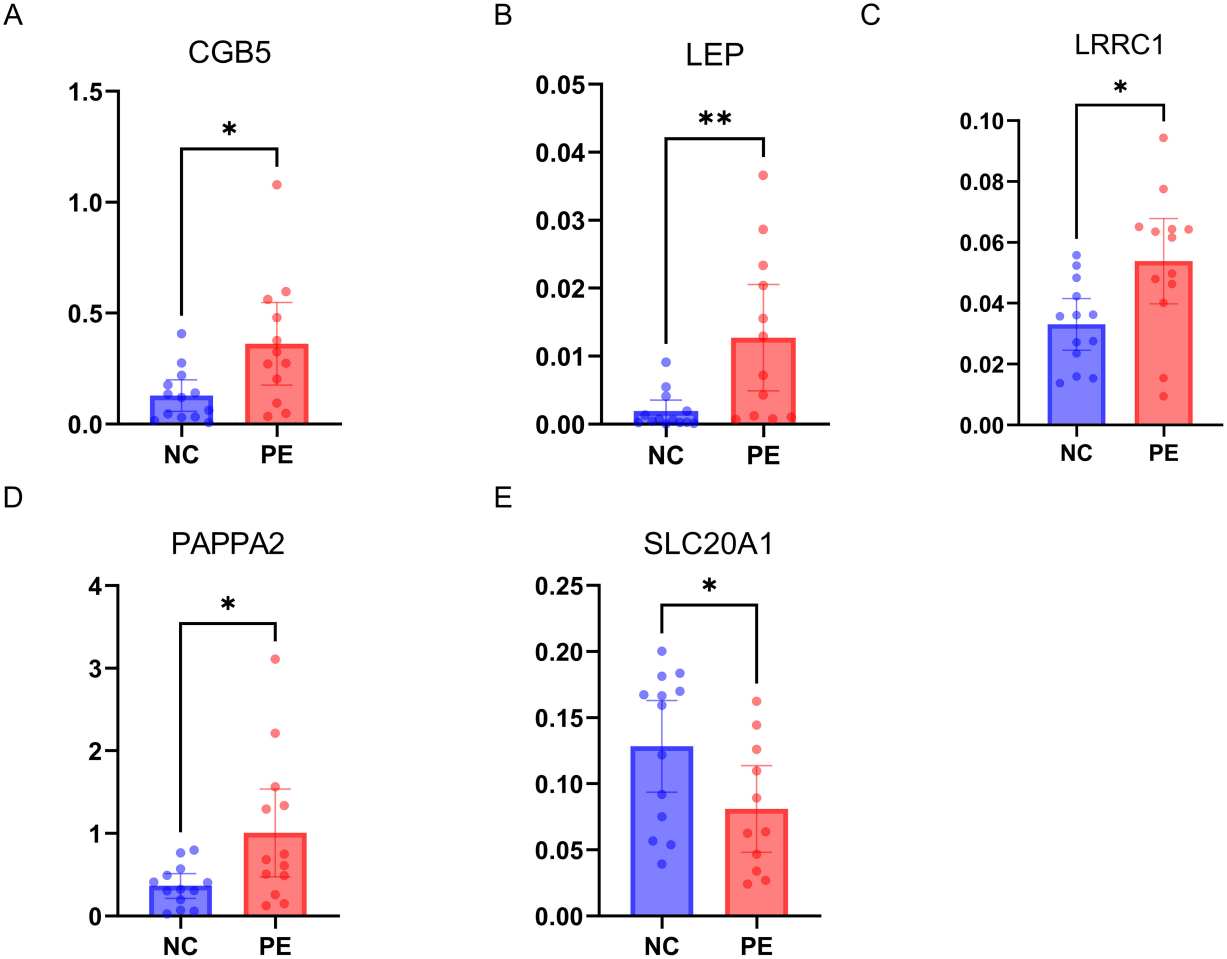
Experimental Validation of 5 Hub Genes by qRT-PCR. **(A–E)** mRNA expressions of 5 hub genes in PE patients (n=12) versus healthy controls (n=13); PE, preeclampsia. * p < 0.05, ** p < 0.01.

The qRT-PCR validation of five hub genes utilizing placental mRNA from 13 healthy controls and 12 PE subjects revealed distinct expression patterns among the tested genes. Significantly elevated expression levels of CGB5, LEP, PAPPA2, LRRC1, and SLC20A1 were observed in the PE group compared to the non-PE group, underscoring their potential roles in the pathogenesis of PE. (**Figures 11A–E**).

## 4 Discussion

Currently, there is no effective treatment for preeclampsia; symptoms generally alleviate only after the delivery of the placenta. Therefore, the prediction and prevention of preeclampsia are of paramount importance (20). In this study, the significant correlation of five key genes (*CGB5, LEP, PAPPA2, LRRC1, SLC20A1*) with PE was established using LASSO, Random Forest, and SVM-RFE techniques. A nomogram incorporating these genes showed strong predictive accuracy for LOPE, while a deep learning model based on them demonstrated superior diagnostic efficacy. qRT-PCR analysis on clinical placenta samples indicated notable expression differences for *CGB5, LEP, PAPPA2, LRRC1, and SLC20A1* between PE patients and healthy controls, underscoring their potential role in PE pathogenesis.

Multiple studies have employed the GEO database to investigate genes involved in the development and diagnosis of PE (21–24). Merging data from various GEO datasets can enhance sample size cost-effectively, but it requires meticulous attention to methodological details (e.g., the probe intensity algorithms) to avoid compromising results. In this study, training data from similar platforms, specifically GPL10558, were merged to ensure consistency in gene expression analysis. The research highlights the importance of adequate sample size and external validation with clinical samples to maintain the biological relevance of gene expression data, emphasizing the integration of prior knowledge to ensure scientific robustness and clinical relevance of the findings.

Using qRT-PCR, we detected differential mRNA expression levels of five hub genes across samples from 13 control and 12 PE-affected placentas. *CGB5*, a gene encoding a subunit of human chorionic gonadotropin (hCG), has been implicated in the pathogenesis of preeclampsia (25, 26). hCG plays a multifaceted role in pregnancy, influencing both immune regulation and angiogenesis. At the maternal-fetal interface, hCG contributes to immune tolerance by attracting Tregs to areas of hCG production, thus maintaining fetal protection against inflammatory responses (27). Tregs, which increase early in pregnancy and peak during the second trimester, are critical for this immune modulation. Dysregulation of hCG may impair this immune tolerance, potentially contributing to pregnancy complications such as PE. In addition to its role in immune regulation, hCG is recognized as an angiogenic factor, impacting endovascular interactions between trophoblasts, endothelial cells, and NK cells (28). NK cells are essential for spiral artery remodeling and trophoblast invasion, and their angiogenic activity is supported by hCG. Studies have shown that hCG stimulates uNK cell proliferation through the mannose receptor, enhancing their non-cytotoxic activity and promoting endovascular processes (29). Disruption in hCG’s regulatory functions could therefore impact both immune tolerance and vascular remodeling, potentially contributing to the development of preeclampsia. Previous research delineated a significant downregulation of Metastasis-associated protein 3 (*MTA3*) in the placenta affected by preeclampsia, in contrast to the upregulation of both *CGB5* and Snail. These findings suggest a critical role for *MTA3* in the repression of hCG and Snail within the placental trophoblast, with its dysregulation being associated with the onset of preeclampsia. Further advancements in this field were made by a recent study, which identified an inhibitory YAP-TEAD4 complex containing the histone methyltransferase EZH2 within the genomic regions of *CGB5* and *CGB7* in the syncytiotrophoblast (STB). This complex is instrumental in the maintenance of the human placental trophoblast epithelium. The study underscored the pivotal role of Yes-associated protein (YAP) not only in activating stemness factors but also in directly repressing genes that facilitate trophoblast cell fusion.

The gene *LEP* encodes leptin, a hormone pivotal in regulating proliferation, protein synthesis, invasion, and apoptosis of placental cells. It plays a crucial role in the early stages of pregnancy. One research has shown that leptin expression is notably increased in the placenta of preeclamptic patients, suggesting its contribution to the disease’s pathogenesis (21). Systematic reviews also have confirmed that preeclampsia is linked to elevated levels of leptin and other adipokines throughout all pregnancy trimesters (22). In obese pregnant women, low serum concentrations of adiponectin and leptin in the first trimester have been associated with preeclampsia, indicating a potential role of adipokine dysregulation in its development (30, 31). Experimental models have further demonstrated that leptin infusion can induce characteristics of clinical preeclampsia in mice, which can be mitigated by specific receptor deletions (32). Chen et al. found that elevated *LEP* levels correlate positively with gamma delta T cells, M0 macrophages, memory B cells, and regulatory T cells, indicating its involvement in immune modulation. Conversely, *LEP* shows negative correlations with resting CD4 memory T cells and activated NK cells (33). These associations suggest that *LEP* contributes to the immune cell imbalance seen in PE, influencing disease mechanisms. *LEP*’s role in angiogenesis and its impact on PE is complex and appears to be context dependent. While leptin is generally recognized as a pro-angiogenic factor, promoting VSMC proliferation through pathways such as phosphatidylinositol-3-kinase (*PI3K*)-protein kinase B (*Akt*)-mammalian target of rapamycin (*mTOR*), the evidence is not entirely consistent (34). Some research indicates potential anti-angiogenic effects (35). This discrepancy highlights the need for further research to clarify leptin’s dual effects during pregnancy. Direct experiments focusing on leptin’s impact on angiogenesis specifically within the context of PE are essential to better understand its role in placentation and the development of this condition.

Recent researches on *PAPPA2*, Pregnancy-Associated Plasma Protein A2, have provided new insights into its role in preeclampsia. *PAPPA2* has shown promise as a potential biomarker for predicting preeclampsia, with good classification performance in identifying women at risk. This protein, localized to differentiated trophoblasts, impairs trophoblast migration, invasion, and network formation *in vitro* by inhibiting epithelial–mesenchymal transition through the downregulation of the Hedgehog signaling pathway (36). Further, hypoxia, a common feature in PE, significantly increases *PAPPA2* expression, which could contribute to poor placentation and impaired angiogenesis. By restraining trophoblast cell functions and affecting placental development, elevated *PAPPA2* may exacerbate inadequate vascular remodeling, leading to the development of PE (37). Interestingly, *PAPPA2* deficiency in mouse models did not exacerbate intrauterine growth restriction, suggesting its upregulation in preeclampsia might not be a compensatory mechanism for impaired fetal growth (38). These findings highlight the multifaceted role of *PAPPA2* in pregnancy and its potential utility in diagnosing and understanding the pathophysiology of preeclampsia.

Recent research on *SLC20A1* (the phosphate transporter PiT1) has illuminated its complex role in various biological processes, yet its direct connection to preeclampsia has not been fully explored (39). PiT1 is known for its involvement in the transport of inorganic phosphate, along with additional roles in regulating TNFα-induced apoptosis, erythropoiesis, cell proliferation, and insulin signaling (40). Its critical function in maintaining endoplasmic reticulum homeostasis and chondrocyte survival during skeletal development has been documented, as well as its significance in sustaining physiological phosphate balance, particularly in kidney function and soft tissue calcification (41). Importantly, PiT1 is crucial for chorioallantois placental morphogenesis, facilitating phosphate symport into syncytiotrophoblast cells, which is essential for their differentiation and function at the maternal-fetal interface (42). Previous study found that *SLC20A2* deficiency in mice lead to fetal growth restriction and pregnancy complications, highlighting its crucial role in placental function. Abnormalities such as altered vascular structure, increased basement membrane deposition, and calcification in *SLC20A2*-deficient placentas suggest impaired angiogenesis and placental development. Similarly, reduced levels of *SLC20A1* and *SLC20A2* in human placentas have been linked to early-onset preeclampsia, indicating that disruptions in these proteins can affect immune regulation and angiogenesis (43). The association between preterm placental calcification and poor pregnancy outcomes further supports the involvement of *SLC20A1* in the pathogenesis of PE through its effects on vascular remodeling.

*LRRC1*, as a constituent of the LAP protein family, plays an essential role in the establishment and maintenance of apical-basal cell polarity (44, 45). Alterations in cell adhesion and polarity are crucial for the transformation of the trophoblast layer, a process integral to placental formation (46). This transformation facilitates the proper invasion of trophoblast cells into the maternal endometrium, a critical step for successful implantation and placental development. Such processes are especially significant in conditions like preeclampsia, where there is a notable reduction in the invasive capability of trophoblast cells (47). Consequently, elevated expression levels of *LRRC1* may impede the cellular rearrangements necessary for modulating polarity, thereby influencing the pathogenesis of preeclampsia. Further studies are needed to elucidate *LRRC1* ‘s precise role and therapeutic potential in preeclampsia.

Immune cells are crucial for supporting embryo implantation and forming the maternal-fetal interface, with immune dysfunction and inflammation being key factors in the development of PE (48, 49). Disruptions in the complex immune environment of pregnancy can lead to pro-inflammatory reactions, oxidative stress, and endothelial dysfunction, potentially contributing to preeclampsia and adverse outcomes (48, 50). Our study expands on previous research by analyzing 28 immune cell types using ssGSEA, revealing notable changes in 23 types in the decidua of PE patients. We observed a strong correlation between five hub genes and regulatory T cells, NK cells, and MDSCs, underscoring their roles in preeclampsia’s pathogenesis (51). We observed a significant reduction in the infiltration of innate immune cells, particularly MDSC, in PE. This observation is consistent with previous studies that suggest MDSC depletion may contribute to pregnancy loss by increasing the cytotoxicity of dNK (decidual natural killer) cells (52). However, the mechanism underlying the connection between MDSC depletion and preeclampsia remains to be fully elucidated. The correlation map reveals a negative association between MDSC and all types of NK cells, hinting at a potential mechanism. These findings suggest that the identified genes play a crucial role in the immune dysfunction associated with PE.

Spiral artery (SA) remodeling is essential for establishing and maintaining a healthy pregnancy, and recent findings highlight the role of NK cells in this process. NK cells, which are more prevalent in the first trimester compared to term pregnancy, play a pivotal role in both promoting SA remodeling and regulating trophoblast invasion through regulating the production of growth factors and cytokines (53). NK cells interact directly or indirectly with EVTs, facilitating the induction and maintenance of immune tolerance, protecting the placenta from infections, and supporting SA remodeling (54). Robson et al. demonstrated that NK cells from early human pregnancies can induce morphological changes in vascular smooth muscle cells (VSMCs) and the breakdown of extracellular matrix components, which are critical for effective remodeling (55). Disruptions in these processes, potentially caused by abnormalities in NK cell function, could contribute to impaired SA remodeling and subsequently lead to the development of preeclampsia.

T cells, including regulatory T cells (Tregs) and effector T cells, are crucial for maintaining immune tolerance and supporting trophoblast function during pregnancy. In preeclampsia, an imbalance between pro-inflammatory T cells and Tregs can disrupt trophoblast function and lead to abnormal placentation. Tregs, essential for pregnancy tolerance, are often compromised in PE, with their insufficiency observed before symptom onset. Studies indicate that sufficient Treg numbers are necessary for proper maternal vascular adaptation and prevention of placental inflammation (56). Imbalances favoring pro-inflammatory CD4+ T cells contribute to inadequate spiral artery remodeling and oxidative stress, which trigger PE symptoms. Immunomodulatory therapies targeting these T cells, such as monoclonal antibodies against TNF-α, IL-17, and IL-6, and adoptive Treg therapy, show promise. These treatments may reduce inflammation, stimulate Tregs, and improve vascular remodeling and placentation, offering potential strategies to prevent or manage preeclampsia effectively (57). To validate the roles of NK and T cells in preeclampsia, further experimental studies are recommended. Flow cytometry can be employed to directly analyze immune cell subsets in blood samples and placentas from preeclamptic patients. Additionally, exploring these mechanisms in animal models and using advanced techniques such single-cell RNA sequencing and spatial multi-omics will provide more detailed insights into the immune dysregulation observed in preeclampsia.

Current predictive models for PE typically integrate clinical characteristics with biomarkers indicative of placental trophoblast cell dysfunction and vascular endothelial damage, including vascular endothelial growth factor (VEGF), soluble tyrosine kinase 1 (sFlt-1), soluble endoglin (sEng), and placental growth factor (PLGF) (58–60). These models are often enhanced with ultrasound measurements of uterine artery blood flow resistance (61, 62). A large study conducted across two maternity hospitals in the UK combined first-trimester maternal demographic characteristics, medical history, and biomarkers using an artificial neural network (ANN), achieving an AUC of 0.770 when using maternal factors alone, and 0.817 when biomarkers were included (62). Moreover, a recent study employed plasma cell-free RNA (cfRNA) signatures to achieve noninvasive PE prediction (63). The preterm PE prediction model achieved an AUC of 0.81 in an independent validation cohort (preterm, n=46; control, n=151). The early-onset PE prediction model had an AUC of 0.88 in an external validation cohort (early-onset PE, n=28; control, n=234). cfRNA holds significant promise for clinical translation due to its noninvasive nature and potential for widespread application in prenatal screening.

Thus, another limitation of the study is the challenge of translating placental-derived biomarkers into early pregnancy screening tools. The potential of using liquid biopsy techniques to analyze circulating cell-free RNA (cfRNA) and DNA (cfDNA) for early prediction of preeclampsia has garnered significant interest. The biomarkers *CGB5*, *LEP*, *LRRC1*, *PAPPA2*, and *SLC20A1* offer varying prospects for predicting preeclampsia. CGB5, a component of hCG, is already used in standard pregnancy tests, making its detection in cfRNA or cfDNA feasible (64). Leptin, involved in metabolic regulation, has been implicated in pregnancy complications. Elevated leptin levels have been observed in some preeclampsia cases, suggesting its potential as a predictive marker (65). *PAPPA2*, a member of the *PAPPA* family, has established roles in pregnancy and is associated with various pregnancy-related conditions. Kramer et al. found that elevated levels of *PAPPA2* are observed in both maternal serum and placental tissue in pregnancies affected by preeclampsia (66). Conversely, the roles of *LRRC1* and *SLC20A1* in preeclampsia remain unclear, necessitating further research. Investigating their expression in cfRNA or cfDNA during early pregnancy is essential to determine their potential viability as screening markers. Developing liquid biopsy assays for these biomarkers poses technical challenges, including the low abundance of cfRNA and cfDNA, the need for highly sensitive and specific detection methods, and the ability to accurately quantify biomarker levels. While techniques such as digital PCR and next-generation sequencing (NGS) are advancing, further optimization is required before they can be implemented in routine prenatal care. In conclusion, while promising, future studies must explore the feasibility of detecting these placental-derived biomarkers in maternal blood during early pregnancy. This would provide a minimally invasive screening tool for preeclampsia, allowing for earlier intervention and better management of the condition.

Another limitation of our study is the inability to strictly differentiate between EOPE and LOPE. Based on the timing of onset, preeclampsia is classified into early-onset PE (EOPE), occurring before 34 weeks of gestation, and late-onset PE (LOPE), which manifests after 34 weeks. Clinically, there is considerable heterogeneity among PE patients, with variations in clinical presentation, pathophysiological mechanisms, and drug responsiveness. EOPE is typically associated with more severe clinical manifestations, and significant progress has been made in recent years regarding its underlying mechanisms (67). However, LOPE is more prevalent than EOPE, and few biomarkers for its early diagnosis have been identified. In our study, we were unable to find a public database with a large sample size and unified sequencing platform that specifically claims to detect LOPE. Expanding our study to include such datasets in the future could potentially address this limitation and further improve our understanding of PE, potentially providing more tailored and accurate predictive tools for these distinct clinical phenotypes.

A further shortcoming of our study is that the methods used to identify key genes through machine learning and deep learning could be further refined. With advancements in deep learning algorithms, the analysis of large-scale multi-omics data has become feasible (68–71). In multi-omics, entities are often interconnected, as seen in genomics, where genes typically function within networks. Graph data, which represent complex relationships and logical rules between nodes, can be analyzed using bioinformatics approaches such as WGCNA and Protein-Protein Interaction (PPI) networks (72, 73). Integrating these approaches with deep learning methods, such as Graph Convolutional Networks (GCN) and Graph Attention Networks (GAT), could offer promising insights. While these deep learning frameworks have shown effectiveness in other diseases, their application in PE prediction remains underexplored and warrants further investigation.

## Data Availability

The original contributions presented in the study are included in the article/**Supplementary Material**. Further inquiries can be directed to the corresponding author.

## References

[1] Dimitriadis E Rolnik DL Zhou W Estrada-Gutierrez G Koga K Francisco RPV. Pre-eclampsia. Nature reviews Disease primers. Nat Rev Dis Primers (2023) 9(1):8. doi: 10.1038/s41572-023-00417-6 36797292

[2] Ives CW Sinkey R Rajapreyar I Tita ATN Oparil S. Preeclampsia-pathophysiology and clinical presentations: JACC state-of-the-art review. J Am Coll Cardiol. (2020) 76:1690–702. doi: 10.1016/j.jacc.2020.08.014 33004135

[3] Yang Y Hua Y Zheng H Jia R Ye Z Su G. Biomarkers prediction and immune landscape in ulcerative colitis: Findings based on bioinformatics and machine learning. Comput Biol Med. (2024) 168:107778. doi: 10.1016/j.compbiomed.2023.107778 38070204

[4] Zhang Z Wang S Zhu Z Nie B. Identification of potential feature genes in non-alcoholic fatty liver disease using bioinformatics analysis and machine learning strategies. Comput Biol Med. (2023) 157:106724. doi: 10.1016/j.compbiomed.2023.106724 36898287

[5] Lai ZZ Zhang J Zhou WJ Shi JW Yang HL Yang SL. Identification of potential biomarkers and immune infiltration characteristics in recurrent implantation failure using bioinformatics analysis. Front Immunol. (2023) 14:992765. doi: 10.3389/fimmu.2023.992765 36776897 PMC9909740

[6] Laub V Devraj K Elias L Schulte D. Bioinformatics for wet-lab scientists: practical application in sequencing analysis. BMC Genomics. (2023) 24:382. doi: 10.1186/s12864-023-09454-7 37420172 PMC10326960

[7] Barrett T Wilhite SE Ledoux P Evangelista C Kim IF Tomashevsky M. NCBI GEO: archive for functional genomics data sets–update. Nucleic Acids Res. (2013) 41:D991–5. doi: 10.1093/nar/gks1193 PMC3531084 23193258

[8] Auslander N Gussow AB Koonin EV. Incorporating machine learning into established bioinformatics frameworks. Int J Mol Sci. (2021) 22(6):2903. doi: 10.3390/ijms22062903 33809353 PMC8000113

[9] Cho YR Kang M. Interpretable machine learning in bioinformatics. Methods (San Diego Calif). (2020) 179:1–2. doi: 10.1016/j.ymeth.2020.05.024 32479800

[10] Gao Y Sun F. Batch normalization followed by merging is powerful for phenotype prediction integrating multiple heterogeneous studies. PloS Comput Biol. (2023) 19:e1010608. doi: 10.1371/journal.pcbi.1010608 37844077 PMC10602384

[11] Langfelder P Horvath S. WGCNA: an R package for weighted correlation network analysis. BMC Bioinf. (2008) 9:559. doi: 10.1186/1471-2105-9-559 PMC2631488 19114008

[12] Kanehisa M Goto S. KEGG: kyoto encyclopedia of genes and genomes. Nucleic Acids Res. (2000) 28:27–30. doi: 10.1093/nar/28.1.27 10592173 PMC102409

[13] Yu G Wang LG Han Y He QY. clusterProfiler: an R package for comparing biological themes among gene clusters. Omics: J Integr Biol. (2012) 16:284–7. doi: 10.1089/omi.2011.0118 PMC3339379 22455463

[14] Engebretsen S Bohlin J. Statistical predictions with glmnet. Clin epigenetics. (2019) 11:123. doi: 10.1186/s13148-019-0730-1 31443682 PMC6708235

[15] Chen X Ishwaran H. Random forests for genomic data analysis. Genomics. (2012) 99:323–9. doi: 10.1016/j.ygeno.2012.04.003 PMC3387489 22546560

[16] Sanz H Valim C Vegas E Oller JM Reverter F. SVM-RFE: selection and visualization of the most relevant features through non-linear kernels. BMC Bioinf. (2018) 19:432. doi: 10.1186/s12859-018-2451-4 PMC6245920 30453885

[17] Shariat SF Capitanio U Jeldres C Karakiewicz PI. Can nomograms be superior to other prediction tools? BJU Int. (2009) 103:492–5. doi: 10.1111/j.1464-410X.2008.08073.x 18990135

[18] Mandrekar JN. Receiver operating characteristic curve in diagnostic test assessment. J Thorac oncology: Off Publ Int Assoc Study Lung Cancer. (2010) 5:1315–6. doi: 10.1097/JTO.0b013e3181ec173d 20736804

[19] Hänzelmann S Castelo R Guinney J. GSVA: gene set variation analysis for microarray and RNA-seq data. BMC Bioinf. (2013) 14:7. doi: 10.1186/1471-2105-14-7 PMC3618321 23323831

[20] Rana S Lemoine E Granger JP Karumanchi SA. Preeclampsia: pathophysiology, challenges, and perspectives. Circ Res. (2019) 124:1094–112. doi: 10.1161/circresaha.118.313276 30920918

[21] Wang Y Bai X Guo X Gao X Chen Y Li H. Bioinformatics analysis combined with clinical sample screening reveals that leptin may be a biomarker of preeclampsia. Front Physiol. (2022) 13:1031950. doi: 10.3389/fphys.2022.1031950 36685185 PMC9846503

[22] Mohamad MA Mohd Manzor NF Zulkifli NF Zainal N Hayati AR Ahmad Asnawi AW. A review of candidate genes and pathways in preeclampsia-an integrated bioinformatical analysis. Biology. (2020) 9(4):62. doi: 10.3390/biology9040062 32230784 PMC7235730

[23] Liu Y Lu X Zhang Y Liu M. Identification and validation of a five-gene diagnostic signature for preeclampsia. Front Genet. (2022) 13:910556. doi: 10.3389/fgene.2022.910556 35774506 PMC9237423

[24] Meng Y Li C Liu CX. Immune cell infiltration landscape and immune marker molecular typing in preeclampsia. Bioengineered. (2021) 12:540–54. doi: 10.1080/21655979.2021.1875707 PMC8806319 33535891

[25] Chen Y Miyazaki J Nishizawa H Kurahashi H Leach R Wang K. MTA3 regulates CGB5 and Snail genes in trophoblast. Biochem Biophys Res Commun. (2013) 433:379–84. doi: 10.1016/j.bbrc.2013.02.102 PMC3761375 23510993

[26] Meinhardt G Haider S Kunihs V Saleh L Pollheimer J Fiala C. Pivotal role of the transcriptional co-activator YAP in trophoblast stemness of the developing human placenta. Proc Natl Acad Sci United States America. (2020) 117:13562–70. doi: 10.1073/pnas.2002630117 PMC7306800 32482863

[27] Schumacher A Brachwitz N Sohr S Engeland K Langwisch S Dolaptchieva M. Human chorionic gonadotropin attracts regulatory T cells into the fetal-maternal interface during early human pregnancy. J Immunol (Baltimore Md: 1950). (2009) 182:5488–97. doi: 10.4049/jimmunol.0803177 19380797

[28] Berndt S Perrier d'Hauterive S Blacher S Péqueux C Lorquet S Munaut C. Angiogenic activity of human chorionic gonadotropin through LH receptor activation on endothelial and epithelial cells of the endometrium. FASEB J. (2006) 20:2630–2. doi: 10.1096/fj.06-5885fje 17065221

[29] Croy BA Esadeg S Chantakru S van den Heuvel M Paffaro VA He H. Update on pathways regulating the activation of uterine Natural Killer cells, their interactions with decidual spiral arteries and homing of their precursors to the uterus. J Reprod Immunol. (2003) 59:175–91. doi: 10.1016/s0165-0378(03)00046-9 12896821

[30] Thagaard IN Hedley PL Holm JC Lange T Larsen T Krebs L. Leptin and Adiponectin as markers for preeclampsia in obese pregnant women, a cohort study. Pregnancy hypertension. (2019) 15:78–83. doi: 10.1016/j.preghy.2018.12.002 30825932

[31] Taylor BD Ness RB Olsen J Hougaard DM Skogstrand K Roberts JM. Serum leptin measured in early pregnancy is higher in women with preeclampsia compared with normotensive pregnant women. Hypertension (Dallas Tex: 1979). (2015) 65:594–9. doi: 10.1161/hypertensionaha.114.03979 PMC4326535 25510827

[32] Faulkner JL Wright D Antonova G Jaffe IZ Kennard S Belin de Chantemèle EJ. Midgestation leptin infusion induces characteristics of clinical preeclampsia in mice, which is ablated by endothelial mineralocorticoid receptor deletion. Hypertension (Dallas Tex: 1979). (2022) 79:1536–47. doi: 10.1161/hypertensionaha.121.18832 PMC9177735 35510543

[33] Chen S Ke Y Chen W Wu S Zhuang X Lin Q. Association of the LEP gene with immune infiltration as a diagnostic biomarker in preeclampsia. Front Mol Biosci. (2023) 10:1209144. doi: 10.3389/fmolb.2023.1209144 37635936 PMC10448764

[34] Shan J Nguyen TB Totary-Jain H Dansky H Marx SO Marks AR. Leptin-enhanced neointimal hyperplasia is reduced by mTOR and PI3K inhibitors. Proc Natl Acad Sci United States America. (2008) 105:19006–11. doi: 10.1073/pnas.0809743105 PMC2585045 19020099

[35] Rodríguez A Gómez-Ambrosi J Catalán V Fortuño A Frühbeck G. Leptin inhibits the proliferation of vascular smooth muscle cells induced by angiotensin II through nitric oxide-dependent mechanisms. Mediators Inflammation. (2010) 2010:105489. doi: 10.1155/2010/105489 PMC2879542 20592755

[36] Chen X Chen K Feng Y Ren C Li W Xiao J. The potential role of pregnancy-associated plasma protein-A2 in angiogenesis and development of preeclampsia. Hypertension research: Off J Japanese Soc Hypertension. (2019) 42:970–80. doi: 10.1038/s41440-019-0224-8 30816319

[37] Wagner PK Otomo A Christians JK. Regulation of pregnancy-associated plasma protein A2 (PAPPA2) in a human placental trophoblast cell line (BeWo). Reprod Biol endocrinology: RB&E. (2011) 9:48. doi: 10.1186/1477-7827-9-48 21496272 PMC3096916

[38] Christians JK Lennie KI Huicochea Munoz MF Binning N. PAPP-A2 deficiency does not exacerbate the phenotype of a mouse model of intrauterine growth restriction. Reprod Biol endocrinology: RB&E. (2018) 16:58. doi: 10.1186/s12958-018-0376-4 29895300 PMC5996520

[39] Rieke JM Zhang R Braun D Yilmaz Ö Japp AS Lopes FM. SLC20A1 is involved in urinary tract and urorectal development. Front Cell Dev Biol. (2020) 8:567. doi: 10.3389/fcell.2020.00567 32850778 PMC7426641

[40] Koumakis E Millet-Botti J Benna JE Leroy C Boitez V Codogno P. Novel function of PiT1/SLC20A1 in LPS-related inflammation and wound healing. Sci Rep. (2019) 9:1808. doi: 10.1038/s41598-018-37551-1 30755642 PMC6372663

[41] Kulesza T Piwkowska A. The impact of type III sodium-dependent phosphate transporters (Pit 1 and Pit 2) on podocyte and kidney function. J Cell Physiol. (2021) 236:7176–85. doi: 10.1002/jcp.30368 33738792

[42] Correia-Branco A Mei A Pillai S Jayaraman N Sharma R Paquette AG. SLC20a1/PiT-1 is required for chorioallantoic placental morphogenesis. Vasc Biol. (2023) 5(1):e220018. doi: 10.1530/vb-22-0018 36795703 PMC10160536

[43] Wallingford MC Gammill HS Giachelli CM. Slc20a2 deficiency results in fetal growth restriction and placental calcification associated with thickened basement membranes and novel CD13 and lamininα1 expressing cells. Reprod Biol. (2016) 16:13–26. doi: 10.1016/j.repbio.2015.12.004 26952749 PMC4841690

[44] Saito H Santoni MJ Arsanto JP Jaulin-Bastard F Le Bivic A Marchetto S. Lano, a novel LAP protein directly connected to MAGUK proteins in epithelial cells. J Biol Chem. (2001) 276:32051–5. doi: 10.1074/jbc.C100330200 11440998

[45] Wang Y Li X Guan X Song Z Liu H Guan Z. The upregulation of leucine-rich repeat containing 1 expression activates hepatic stellate cells and promotes liver fibrosis by stabilizing phosphorylated smad2/3. Int J Mol Sci. (2024) 25:2735. doi: 10.3390/ijms25052735 38473980 PMC10932271

[46] Boss AL Chamley LW James JL. Placental formation in early pregnancy: how is the centre of the placenta made? Hum Reprod Update. (2018) 24:750–60. doi: 10.1093/humupd/dmy030 30257012

[47] Fisher SJ. Why is placentation abnormal in preeclampsia? Am J obstetrics gynecology. (2015) 213(4 Suppl):S115–S122. doi: 10.1016/j.ajog.2015.08.042 PMC4592742 26428489

[48] Aneman I Pienaar D Suvakov S Simic TP Garovic VD McClements L. Mechanisms of key innate immune cells in early- and late-onset preeclampsia. Front Immunol. (2020) 11:1864. doi: 10.3389/fimmu.2020.01864 33013837 PMC7462000

[49] Han X Ghaemi MS Ando K Peterson LS Ganio EA Tsai AS. Differential dynamics of the maternal immune system in healthy pregnancy and preeclampsia. Front Immunol. (2019) 10:1305. doi: 10.3389/fimmu.2019.01305 31263463 PMC6584811

[50] Opichka MA Rappelt MW Gutterman DD Grobe JL McIntosh JJ. Vascular dysfunction in preeclampsia. Cells. (2021) 10(11):3055. doi: 10.3390/cells10113055 34831277 PMC8616535

[51] Deer E Herrock O Campbell N Cornelius D Fitzgerald S Amaral LM. The role of immune cells and mediators in preeclampsia. Nat Rev Nephrol. (2023) 19:257–70. doi: 10.1038/s41581-022-00670-0 PMC10038936 36635411

[52] Ren J Zeng W Tian F Zhang S Wu F Qin X. Myeloid-derived suppressor cells depletion may cause pregnancy loss via upregulating the cytotoxicity of decidual natural killer cells. Am J Reprod Immunol. (2019) 81:e13099. doi: 10.1111/aji.13099 30737988

[53] Hanna J Goldman-Wohl D Hamani Y Avraham I Greenfield C Natanson-Yaron S. Decidual NK cells regulate key developmental processes at the human fetal-maternal interface. Nat Med. (2006) 12:1065–74. doi: 10.1038/nm1452 16892062

[54] Wei XW Zhang YC Wu F Tian FJ Lin Y. The role of extravillous trophoblasts and uterine NK cells in vascular remodeling during pregnancy. Front Immunol. (2022) 13:951482. doi: 10.3389/fimmu.2022.951482 37408837 PMC10319396

[55] Robson A Harris LK Innes BA Lash GE Aljunaidy MM Aplin JD. Uterine natural killer cells initiate spiral artery remodeling in human pregnancy. FASEB J. (2012) 26:4876–85. doi: 10.1096/fj.12-210310 22919072

[56] Robertson SA Green ES Care AS Moldenhauer LM Prins JR Hull ML. Therapeutic potential of regulatory T cells in preeclampsia-opportunities and challenges. Front Immunol. (2019) 10:478. doi: 10.3389/fimmu.2019.00478 30984163 PMC6448013

[57] Murray EJ Gumusoglu SB Santillan DA Santillan MK. Manipulating CD4+ T cell pathways to prevent preeclampsia. Front Bioeng Biotechnol. (2021) 9:811417. doi: 10.3389/fbioe.2021.811417 35096797 PMC8789650

[58] Loftness BC Bernstein I McBride CA Cheney N McGinnis EW McGinnis RS. Preterm preeclampsia risk modelling: examining hemodynamic, biochemical, and biophysical markers prior to pregnancy. MedRxiv: Preprint Server Health Sci. (2023) 06:06. doi: 10.1101/2023.02.28.23286590 38083443

[59] Melinte-Popescu AS Vasilache IA Socolov D Melinte-Popescu M. Predictive performance of machine learning-based methods for the prediction of preeclampsia-A prospective study. J Clin Med. (2023) 12:4. doi: 10.3390/jcm12020418 PMC9865606 36675347

[60] Xue Y Yang N Gu X Wang Y Zhang H Jia K. Risk prediction model of early-onset preeclampsia based on risk factors and routine laboratory indicators. Life. (2023) 13:28. doi: 10.3390/life13081648 PMC10455518 37629504

[61] Gomez-Jemes L Madalina Oprescu A Chimenea-Toscano A Garcia-Diaz L Romero-Ternero M. Machine learning to predict pre-eclampsia and intrauterine growth restriction in pregnant women. Electronics. (2022) 11(19):3240. doi: 10.3390/electronics11193240

[62] Ansbacher-Feldman Z Syngelaki A Meiri H Cirkin R Nicolaides KH Louzoun Y. Machine-learning-based prediction of pre-eclampsia using first-trimester maternal characteristics and biomarkers. Ultrasound Obstet Gynecol. (2022) 60:739–45. doi: 10.1002/uog.26105 36454636

[63] Zhou S Li J Yang W Xue P Yin Y Wang Y. Noninvasive preeclampsia prediction using plasma cell-free RNA signatures. Am J obstetrics gynecology. (2023) 229:553. doi: 10.1016/j.ajog.2023.05.015 37211139

[64] Barjaktarovic M Korevaar TIM Jaddoe VWV de Rijke YB Peeters RP Steegers EAP. Human chorionic gonadotropin and risk of pre-eclampsia: prospective population-based cohort study. Ultrasound Obstet Gynecol. (2019) 54:477–83. doi: 10.1002/uog.20256 PMC6856821 30834627

[65] Song Y Gao J Qu Y Wang S Wang X Liu J. Serum levels of leptin, adiponectin and resistin in relation to clinical characteristics in normal pregnancy and preeclampsia. Clin Chim Acta. (2016) 458:133–7. doi: 10.1016/j.cca.2016.04.036 27154800

[66] Kramer AW Lamale-Smith LM Winn VD. Differential expression of human placental PAPP-A2 over gestation and in preeclampsia. Placenta. (2016) 37:19–25. doi: 10.1016/j.placenta.2015.11.004 26748159 PMC4848394

[67] Broekhuizen M Hitzerd E van den Bosch TPP Dumas J Verdijk RM van Rijn BB. The placental innate immune system is altered in early-onset preeclampsia, but not in late-onset preeclampsia. Front Immunol. (2021) 12:780043. doi: 10.3389/fimmu.2021.780043 34992598 PMC8724430

[68] Yu L Liu C Yang JYH Yang P. Ensemble deep learning of embeddings for clustering multimodal single-cell omics data. Bioinformatics. (2023) 39(6):btad382. doi: 10.1093/bioinformatics/btad382 37314966 PMC10287920

[69] Poirion OB Jing Z Chaudhary K Huang S Garmire LX. DeepProg: an ensemble of deep-learning and machine-learning models for prognosis prediction using multi-omics data. Genome Med. (2021) 13:112. doi: 10.1186/s13073-021-00930-x 34261540 PMC8281595

[70] Morrow AK Hughes JW Singh J Joseph AD Yosef N. Epitome: predicting epigenetic events in novel cell types with multi-cell deep ensemble learning. Nucleic Acids Res. (2021) 49:e110. doi: 10.1093/nar/gkab676 34379786 PMC8565335

[71] Aybey E Gümüş Ö. SENSDeep: an ensemble deep learning method for protein-protein interaction sites prediction. Interdiscip sciences Comput Life Sci. (2023) 15:55–87. doi: 10.1007/s12539-022-00543-x 36346583

[72] Réau M Renaud N Xue LC Bonvin A. DeepRank-GNN: a graph neural network framework to learn patterns in protein-protein interfaces. Bioinformatics. (2023) 39(1):btac759. doi: 10.1093/bioinformatics/btac759 36420989 PMC9805592

[73] Ciortan M DeFrance M. GNN-based embedding for clustering scRNA-seq data. Bioinformatics. (2022) 38:1037–44. doi: 10.1093/bioinformatics/btab787 34850828

