# Supplementary material for: Development and validation of preeclampsia predictive models using key genes from bioinformatics and machine learning approaches
Source: Front Immunol. 2024 Oct 31;15:1416297. doi: 10.3389/fimmu.2024.1416297 (PMC11560445; doi:10.3389/fimmu.2024.1416297)
Supplement: Supplementary file 1 [file DataSheet1.doc]

*Online Data Supplement*

**Development and Validation of Preeclampsia Predictive Models Using Key Genes from Bioinformatics and Machine Learning Approaches**

Qian Li^1,#^, Xiaowei Wei^1,#^, Fan Wu^2^, Chuanmei Qin^1^, Junpeng Dong^1^, Cailian Chen^3^, Yi Lin^1,*^

1. Reproductive Medicine Center, Shanghai Sixth People's Hospital Affiliated to Shanghai Jiao Tong University School of Medicine, Shanghai, China.

2. The International Peace Maternity and Child Health Hospital Affiliated to Shanghai Jiao Tong University School of Medicine, Shanghai, China.

3. Department of Automation, Shanghai Jiao Tong University, Key Laboratory of System Control and Information Processing, Ministry of Education of China, Shanghai, China.

^#^ Qian Li and Xiaowei Wei contributed equally to this work.

* Corresponding Author: Yi Lin, [yilinonline@126.com](mailto:yilinonline@126.com), Reproductive Medicine Center, Shanghai Sixth People’s Hospital Affiliated to Shanghai Jiao Tong University School of Medicine, Shanghai, China. No. 600 Yishan Road, Xuhui District, Shanghai, China.

# Table S1. Sequences of primer sets used for qRT-PCR

# Table S2: Full list of differentially expressed genes (DEGs).

# Table S3: WGCNA blue module gene list.

# Table S4: WGCNA brown module gene list.

# Table S5: WGCNA grey module gene list.

# Table S6: WGCNA turquoise module gene list.

# Table S7: WGCNA yellow module gene list.

# Figure S1. Top 30 genes based on RF importance scores; RF, Random Forest;

# Code of the Machine-learning-based feature selection models.

# Code of the Machine-learning-based PE risk prediction models.

# Table S1.

| **Gene** | **Forward primer** | **Reverse primer** |
| --- | --- | --- |
| **LEP** | TGCCTTCCAGAAACGTGATCC | CTCTGTGGAGTAGCCTGAAGC |
| **CGB5** | ACCGTCAACACCACCATCTGTG | GAAGCGCACATCGCGGTAGTTG |
| **PAPPA2** | AGAATAAGCCTGGCGATTTTGG | GCCCTTAGGTAGTTCCCAGC |
| **LRRC1** | TGCAACCGTCATGTGGAGAG | CTCCGGGCATAGCGGTAGA |
| **SLC20A1** | GGAAGGGCTTGATTGACGTG | CAGAACCAAACATAGCACTGACT |

# Table S2.

See in supplementary table2_DEGs.csv

# Table S3-S7.

See in supplementary table3-7_WGCNA.zip

# Figure S1.


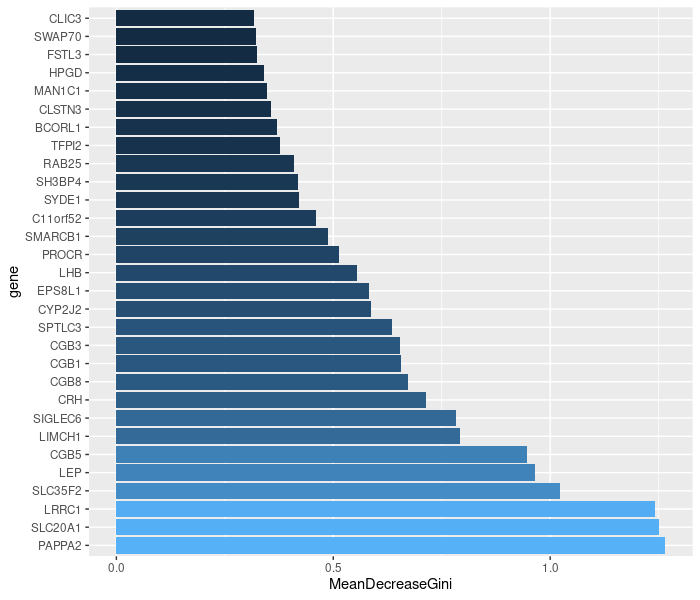


# Code of the Machine-learning-based feature selection models.

library(tidyverse)

library(glmnet)

library(VennDiagram)

library(sigFeature)

library(e1071)

library(caret)

library(kernlab)

library(randomForest)

##################LASSO##################

colnames(WGCNA_DEGs)[1]<-"SYMBOL"

hubgenes=c(WGCNA2_DEGs$SYMBOL)

hubgenes_expression<-exprSet[match(hubgenes,rownames(exprSet)),]

hubgenes_selected=as.matrix(hubgenes_expression[,c(1:ncol(hubgenes_expression))])

hubgenes_selected=t(hubgenes_selected)

hubgenes_selected <- data.frame(X = rownames(hubgenes_selected), hubgenes_selected)

merged_data <- merge(talgroup, hubgenes_selected, by = "X")

rownames(merged_data)<-merged_data$X

merged_data$X <- NULL

train <- merged_data

# write.csv(train,"train.csv")

set.seed(10)

x2 <- as.matrix(train[,-1])

y2 <- train$group

fit2=glmnet(x2,y2,family = "binomial",maxit = 1000)

plot(fit2,xvar="lambda",label = TRUE)

cvfit2 = cv.glmnet(x2,y2,family="binomia",maxit = 1000)

plot(cvfit2)

coef2=coef(fit2,s = cvfit2$lambda.min)

index=which(coef2 != 0)

actCoef2=coef2[index]

lassoGene2=row.names(coef2)[index]

geneCoef2=cbind(Gene=lassoGene2,Coef=actCoef2)

lassoGene2 <- lassoGene2[-1]

actCoef2<- actCoef2[-1]

write.csv(lassoGene2,"WGCNA_DEGs_feature_lasso.csv")

##################SVM-RFE##################

y2 <- as.factor(y2)

y2 <- as.numeric(y2)

Profile=rfe(x=x2, y=y2,

sizes = c(2,4,6,8, seq(10,40,by=3)),

rfeControl = rfeControl(functions = caretFuncs, method = "cv", number = 10),

methods="svmRadial")

featureGenes=Profile$optVariables

variable= Profile$results$Variables

rmse= Profile$results$RMSE

plot(variable, rmse, xlab="Variables", ylab="RMSE (10-fold Cross-Validation)", col="darkgreen")

lines(variable, rmse, col="darkgreen")

wmin=which.min(rmse)

wmin.x=variable[wmin]

wmin.y=rmse[wmin]

points(wmin.x, wmin.y, col="blue", pch=16,font = 10)

text(wmin.x, wmin.y, paste0('N=',wmin.x), pos=2, col=3,font = 10)

##################RF##################

rf <- randomForest(as.factor(group)~., data = train,

ntree = 1000,

nodesize = 10,

splitrule = 'logrank',

importance = T,

proximity = T,

forest = T,

seed = 1234)

plot(rf)

importance <- as.data.frame(rf$importance)

importance <- importance[order(importance$MeanDecreaseGini,

decreasing = TRUE), ]

write.table(importance,file = "importance_class.txt",quote = F,sep = '\t', row.names = T, col.names = T)

varImpPlot(rf,

n.var = min(30, nrow(rf$importance)),

main = 'Top30 - variable importance')

set.seed(315)

train$group <- ifelse(train$group == "PE", 1, 0)

train.cv <- replicate(5, rfcv(train [,-1],

train$group, cv.fold = 10,step = 1.5), simplify = FALSE)

train.cv<-rfcv(train [,-1], train$group, cv.fold = 10,step = 1.5)

train.cv$error.cv

imp = read.table("importance_class.txt", header=T, row.names= 1, sep="\t")

imp$gene<-rownames(imp)

imp=imp[order(imp$MeanDecreaseGini), ]

imp = head(imp, n=30)

imp$gene <- factor(imp$gene, levels = imp$gene)

p=ggplot(data = imp, mapping = aes(x=gene,y=MeanDecreaseGini,fill=MeanDecreaseGini)) +

geom_bar(stat="identity")+coord_flip()+

theme(legend.position = "none")

# Code of the Machine-learning-based PE risk prediction models.

##################Nomogram##################

library(rms)

sig_gene<-c("CGB5","LEP","PAPPA2","LRRC1","SLC20A1")

x <- t(exprSet)

x <- x[,sig_gene]

x <- as.data.frame(x)

x$Status<-talgroup$group

ddist <- datadist(x);

options(datadist='ddist')

model <- lrm(Status ~ ., data = x,x=TRUE,y=TRUE)

nomogram <- nomogram(model, fun = function(x)1/(1+exp(-x)),funlabel="Risk of Event",conf.int=F,lp=F,fun.at=c(.001,.01,.05,seq(.1,.9,by=.2),.95,.99,.99))

grid.newpage()

png(file = "./nomogram.tif", width = 800, height = 700)

plot(nomogram)

dev.off()

cal<-rms::calibrate(model,method = "boot",B=200)

plot(cal,

xlim = c(0,1),

xlab = "Predicted Probability",

ylab="Observed Probability",

legend =FALSE,

subtitles = FALSE)

abline(0,1,col="black",lty=2,lwd=2)

lines(cal[,c("predy","calibrated.orig")],

type="l",lwd=2,col="red",pch=16)

lines(cal[,c("predy","calibrated.corrected")],

type="l",lwd=2,col="blue",pch=16)

legend(0.65,0.55,

c("Ideal","Apparent","Bias-corrected"),

lty = c(2,1,1),

lwd = c(2,1,1),

col = c("black","red","blue"),

bty="n")

v<-validate(model, method="boot", B=1000, dxy=T)

Dxy = v[rownames(v)=="Dxy", colnames(v)=="index.corrected"]

orig_Dxy = v[rownames(v)=="Dxy", colnames(v)=="index.orig"]

bias_corrected_c_index <- abs(Dxy)/2+0.5

orig_c_index <- abs(orig_Dxy)/2+0.5

##################FCNN/MLP (python3)##################

import numpy as np

import matplotlib.pyplot as plt

from sklearn.metrics import roc_curve, auc, roc_auc_score

from sklearn.utils import resample

from sklearn.model_selection import KFold

from tensorflow.keras.models import Sequential

from tensorflow.keras.layers import Dense, Dropout

from tensorflow.keras.optimizers import Adam

from tensorflow.keras.regularizers import l2

from tensorflow.keras.callbacks import EarlyStopping

X_train = myseeddf.iloc[:, 1:6].values

y_train = myseeddf['Status'].factorize()[0]

kf = KFold(n_splits=5, shuffle=True, random_state=42)

train_roc_list = []

val_roc_list = []

train_auc_list = []

val_auc_list = []

train_ci_list = []

val_ci_list = []

early_stop = EarlyStopping(monitor='val_loss', patience=10, restore_best_weights=True)

def bootstrap_auc_ci(y_true, y_pred, n_bootstraps=1000, alpha=0.95):

bootstrapped_scores = []

rng = np.random.RandomState(42)

for _ in range(n_bootstraps):

indices = rng.randint(0, len(y_pred), len(y_pred))

if len(np.unique(y_true[indices])) < 2:

continue

score = roc_auc_score(y_true[indices], y_pred[indices])

bootstrapped_scores.append(score)

sorted_scores = np.array(bootstrapped_scores)

sorted_scores.sort()

ci_lower = sorted_scores[int((1 - alpha) / 2 * len(sorted_scores))]

ci_upper = sorted_scores[int((1 + alpha) / 2 * len(sorted_scores))]

return ci_lower, ci_upper

for train_index, val_index in kf.split(X_train):

X_fold_train, X_fold_val = X_train[train_index], X_train[val_index]

y_fold_train, y_fold_val = y_train[train_index], y_train[val_index]

model = Sequential([

Dense(64, activation='relu', input_shape=(5,), kernel_regularizer=l2(0.001)),

Dropout(0.5),

Dense(1, activation='sigmoid')

])

model.compile(

optimizer=Adam(learning_rate=0.01),

loss='binary_crossentropy',

metrics=['accuracy']

)

model.fit(

X_fold_train, y_fold_train,

epochs=100,

batch_size=32,

validation_data=(X_fold_val, y_fold_val),

callbacks=[early_stop],

verbose=2

)

y_fold_train_pred = model.predict(X_fold_train).ravel()

y_fold_val_pred = model.predict(X_fold_val).ravel()

fpr_train, tpr_train, _ = roc_curve(y_fold_train, y_fold_train_pred)

fpr_val, tpr_val, _ = roc_curve(y_fold_val, y_fold_val_pred)

train_auc = auc(fpr_train, tpr_train)

val_auc = auc(fpr_val, tpr_val)

train_roc_list.append((fpr_train, tpr_train))

val_roc_list.append((fpr_val, tpr_val))

train_auc_list.append(train_auc)

val_auc_list.append(val_auc)

ci_train_lower, ci_train_upper = bootstrap_auc_ci(y_fold_train, y_fold_train_pred)

ci_val_lower, ci_val_upper = bootstrap_auc_ci(y_fold_val, y_fold_val_pred)

train_ci_list.append((ci_train_lower, ci_train_upper))

val_ci_list.append((ci_val_lower, ci_val_upper))

print(f"Fold Train AUC: {train_auc:.4f}, 95% CI: {ci_train_lower:.4f} - {ci_train_upper:.4f}")

print(f"Fold Validation AUC: {val_auc:.4f}, 95% CI: {ci_val_lower:.4f} - {ci_val_upper:.4f}")

plt.figure(figsize=(10, 6))

for i, (fpr, tpr) in enumerate(train_roc_list):

ci_lower, ci_upper = train_ci_list[i]

plt.plot(fpr, tpr, label=f"Fold {i+1} AUC = {train_auc_list[i]:.4f} (95% CI: {ci_lower:.4f} - {ci_upper:.4f})")

plt.plot([0, 1], [0, 1], 'k--', lw=2)

plt.xlabel('False Positive Rate')

plt.ylabel('True Positive Rate')

plt.title('Training Set ROC Curves with AUC and 95% CI')

plt.legend(loc="lower right")

plt.grid(True)

plt.savefig("training_set_roc_curves.png")

plt.show()

plt.figure(figsize=(10, 6))

for i, (fpr, tpr) in enumerate(val_roc_list):

ci_lower, ci_upper = val_ci_list[i]

plt.plot(fpr, tpr, label=f"Fold {i+1} AUC = {val_auc_list[i]:.4f} (95% CI: {ci_lower:.4f} - {ci_upper:.4f})")

plt.plot([0, 1], [0, 1], 'k--', lw=2)

plt.xlabel('False Positive Rate')

plt.ylabel('True Positive Rate')

plt.title('Test Set ROC Curves with AUC and 95% CI')

plt.legend(loc="lower right")

plt.grid(True)

plt.savefig("Test_set_roc_curves.png")

plt.show()
